# Dynamics of disease characteristics and clinical management of critically ill COVID-19 patients over the time course of the pandemic: an analysis of the prospective, international, multicentre RISC-19-ICU registry

**DOI:** 10.1186/s13054-022-04065-2

**Published:** 2022-07-04

**Authors:** Pedro David Wendel-Garcia, André Moser, Marie-Madlen Jeitziner, Hernán Aguirre-Bermeo, Pedro Arias-Sanchez, Janina Apolo, Ferran Roche-Campo, Diego Franch-Llasat, Gian-Reto Kleger, Claudia Schrag, Urs Pietsch, Miodrag Filipovic, Sascha David, Klaus Stahl, Souad Bouaoud, Amel Ouyahia, Patricia Fodor, Pascal Locher, Martin Siegemund, Nuria Zellweger, Sara Cereghetti, Peter Schott, Gianfilippo Gangitano, Maddalena Alessandra Wu, Mario Alfaro-Farias, Gerardo Vizmanos-Lamotte, Hatem Ksouri, Nadine Gehring, Emanuele Rezoagli, Fabrizio Turrini, Herminia Lozano-Gómez, Andrea Carsetti, Raquel Rodríguez-García, Bernd Yuen, Anja Baltussen Weber, Pedro Castro, Jesus Oscar Escos-Orta, Alexander Dullenkopf, Maria C. Martín-Delgado, Theodoros Aslanidis, Marie-Helene Perez, Frank Hillgaertner, Samuele Ceruti, Marilene Franchitti Laurent, Julien Marrel, Riccardo Colombo, Marcus Laube, Alberto Fogagnolo, Michael Studhalter, Tobias Wengenmayer, Emiliano Gamberini, Christian Buerkle, Philipp K. Buehler, Stefanie Keiser, Muhammed Elhadi, Jonathan Montomoli, Philippe Guerci, Thierry Fumeaux, Reto A. Schuepbach, Stephan M. Jakob, Yok-Ai Que, Matthias Peter Hilty, the RISC-19-ICU Investigators, Matthias P. Hilty, Pedro Wendel-Garcia, Reto A. Schuepbach, Jonathan Montomoli, Philippe Guerci, Thierry Fumeaux, Souad Bouaoud, Amel Ouyahia, Meriem Abdoun, Mounira Rais, Mario Alfaro-Farias, Gerardo Vizmanos-Lamotte, Angel Caballero, Thomas Tschoellitsch, Jens Meier, Hernán Aguirre-Bermeo, Pedro Arias-Sanchez, Janina Apolo, Luis A. Martinez, Hugo Tirapé-Castro, Islam Galal, Samar Tharwat, Ibrahim Abdehaleem, Geoffrey Jurkolow, Philippe Guerci, Emmanuel Novy, Marie-Reine Losser, Tobias Wengenmayer, Viviane Zotzmann, Sascha David, Klaus Stahl, Benjamin Seeliger, Tobias Welte, Theodoros Aslanidis, Anita Korsos, Luqman Abdulkhudhur Ahmed, Hashim Talib Hashim, Reza Nikandish, Andrea Carsetti, Erika Casarotta, Paolo Giaccaglia, Emanuele Rezoagli, Matteo Giacomini, Aurora Magliocca, Giuliano Bolondi, Antonella Potalivo, Alberto Fogagnolo, Luca Salvi, Maddalena A. Wu, Chiara Cogliati, Riccardo Colombo, Emanuele Catena, Fabrizio Turrini, Maria S. Simonini, Silvia Fabbri, Jonathan Montomoli, Emiliano Gamberini, Gianfilippo Gangitano, Maria M. Bitondo, Francesca Maciopinto, Enrico de Camillis, Marta Venturi, Maria Grazia Bocci, Massimo Antonelli, Arowa Alansari, Abdurraouf Abusalama, Osama Omar, Muhannud Binnawara, Hind Alameen, Muhammed Elhadi, Abdulmueti Alhadi, Ahmed Arhaym, Diederik Gommers, Can Ince, Mustafa Jayyab, Mohammed Alsharif, Raquel Rodríguez-García, Jorge Gámez-Zapata, Xiana Taboada-Fraga, Pedro Castro, Javier Fernandez, Enric Reverter, Arantxa Lander-Azcona, Jesús Escós-Orta, Maria C. Martín-Delgado, Angela Algaba-Calderon, Ferran Roche-Campo, Diego Franch-Llasat, Pablo Concha, Esther Sauras-Colón, Herminia Lozano-Gómez, Begoña Zalba-Etayo, Maria P. Montes, Marc P. Michot, Alexander Klarer, Rolf Ensner, Peter Schott, Severin Urech, Martin Siegemund, Nuria Zellweger, Caroline E. Gebhard, Alexa Hollinger, Lukas Merki, Adriana Lambert, Marcus Laube, Marie M. Jeitziner, Andre Moser, Yok-Ai Que, Stephan M. Jakob, Jan Wiegand, Bernd Yuen, Barbara Lienhardt-Nobbe, Andrea Westphalen, Petra Salomon, Frank Hillgaertner, Marianne Sieber, Alexander Dullenkopf, Giulio Barana, Hatem Ksouri, Govind O. Sridharan, Sara Cereghetti, Filippo Boroli, Jerome Pugin, Serge Grazioli, Christian Bürkle, Julien Marrel, Mirko Brenni, Isabelle Fleisch, Marie-Helene Perez, Anne-Sylvie Ramelet, Anja Baltussen Weber, Peter Gerecke, Andreas Christ, Samuele Ceruti, Andrea Glotta, Maira Biggiogero, Katharina Marquardt, Tobias Hübner, Thomas Neff, Hermann Redecker, Thierry Fumeaux, Mallory Moret-Bochatay, Marco Betello, Friederike Meyer zu Bentrup, Michael Studhalter, Michael Stephan, Nadine Gehring, Daniela Selz, Gian-Reto Kleger, Claudia Schrag, Urs Pietsch, Miodrag Filipovic, Anette Ristic, Antje Heise, Marilene Franchitti Laurent, Jean-Christophe Laurent, Tomislav Gaspert, Christoph Haberthuer, Patricia Fodor, Pascal Locher, Pedro D. Wendel Garcia, Matthias P. Hilty, Reto Schuepbach, Stefanie Keiser, Dorothea Heuberger, Jan Bartussek, Philipp Bühler, Silvio Brugger, Eva-Maria Kleinert, Kim-Jana Fehlbier, Aghyad Danial, Maher Almousa, Yazan Abdulbaki, Kamil Sannah, Elif Colak, Nandor Marczin, Saba Al-Ameri

**Affiliations:** 1 Institute of Intensive Care Medicine, University Hospital of Zurich, Zurich, Switzerland; 2 The RISC-19-ICU Registry Board, University of Zurich, Zurich, Switzerland; 3 Clinical Trials Unit Bern, University of Bern, Bern, Switzerland; 4 Department of Intensive Care Medicine, University Hospital Bern, Bern, Switzerland; 5 Unidad de Cuidados Intensivos, Hospital Vicente Corral Moscoso, Cuenca, Ecuador; 6 Intensive Care Department, Hospital Verge de La Cinta de Tortosa, Tarragona, Spain; 7 Medizinische Intensivstation, Kantonsspital St. Gallen, St. Gallen, Switzerland; 8 Departement of Anesthesiology and Intensive Care Medicine, Kantonsspital St. Gallen, St. Gallen, Switzerland; 9 Medical Intensive Care, Medical School Hannover, Hannover, Germany; 10 Department of Internal Medicine, Centre Hospitalo Universitaire - Saadna Mohamed Abdnour, Setif, Algeria; 11 Interdisziplinaere Intensivstation, Stadtspital Zurich - Triemli, Zurich, Switzerland; 12 Department Intensivmedizin, Universitaetsspital Basel, Basel, Switzerland; 13 Division of Intensive Care, University Hospitals of Geneva, Geneva, Switzerland; 14 Institut Fuer Anesthaesie Und Intensivmedizin, Zuger Kantonsspital AG, Baar, Switzerland; 15 Department of Emergency Medicine, Ospedale Infermi, Rimini, Italy; 16 Department of Internal Medicine, ASST Fatebenefratelli Sacco - “Luigi Sacco” Hospital, Milan, Italy; 17 Unidad de Cuidados Intensivos, Hospital Nostra Senyora de Meritxell, Escaldes-Engordany, Andorra; 18 Soins Intensifs, Hopital Cantonal de Fribourg, Fribourg, Switzerland; 19 Intensivstation, Kantonsspital Schaffhausen, Schaffhausen, Switzerland; 20 Department of Anesthesia and Intensive Care Medicine, Policlinico San Marco - Gruppo Ospedaliero San Donato, Bergamo, Italy; 21 Department of Medicine and Surgery, University of Milano-Bicocca, Monza, Italy; 22 Internal Medicine, Azienda Ospedaliera Universitaria di Modena, Modena, Italy; 23 Unidad de Cuidados Intensivos, Hospital Clínico Universitario Lozano Blesa, Saragossa, Spain; 24 Anesthesia and Intensive Care Unit, Azienda Ospedaliero Universitaria Ospedali Riuniti di Ancona, Ancona, Italy; 25 Department of Biomedical Sciences and Public Health, Università Politecnica delle Marche, Ancona, Italy; 26 Servicio de Medicina Intensiva, Complejo Hospitalario Universitario A Coruña, A Coruña, Spain; 27 Interdisziplinaere Intensivstation, Spital Buelach, Buelach, Switzerland; 28 Anaesthesie Und Intensivmedizin, Kantonsspital Baselland, Liestal, Switzerland; 29 Medical Intensive Care Unit, Hospital Clínic de Barcelona – IDIBAPS University of Barcelona, Barcelona, Spain; 30 Servicio de Medicina Intensiva, Hospital General San Jorge, Huesca, Spain; 31 Institut Fuer Anaesthesie und Intensivmedizin, Spital Thurgau, Frauenfeld, Switzerland; 32 Servicio de Medicina Intensiva, Hospital Universitario de Torrejon, Madrid, Spain; 33 Intensive Care Unit, St. Paul General Hospital, Thessaloniki, Greece; 34 Pediatric Intensive Care Unit, University Hospital Lausanne, Lausanne, Switzerland; 35 Intensivmedizin, Kantonsspital Graubuenden, Chur, Switzerland; 36 Dipartimento Area Critica, Clinica Luganese Moncucco, Lugano, Switzerland; 37 Service d’Anesthesiologie, EHNV, Yverdon-les-Bains, Switzerland; 38 Institut Für Anaesthesiologie Intensivmedizin & Rettungsmedizin, See-Spital Horgen & Kilchberg, Horgen, Switzerland; 39 Division of Anesthesia and Intensive Care, ASST Fatebenefratelli Sacco - “Luigi Sacco” Hospital, Milan, Italy; 40 Department Intensive Care Medicine, Spitalzentrum Biel, Biel, Switzerland; 41 Anesthesia and Intensive Care, Azienda Ospedaliero-Universitaria di Ferrara, Cona, Italy; 42 Intensivmedizin & Intermediate Care, Kantonsspital Olten, Olten, Switzerland; 43 Department of Medicine III - Interdisciplinary Medical Intensive Care, Medical Center University of Freiburg, Freiburg, Germany; 44 Department of Anesthesia and Intensive Care, Infermi Hospital AUSL - Romagna, Rimini, Italy; 45 Intensivstation, Spital Grabs, Grabs, Switzerland; 46 Faculty of Medicine, University of Tripoli, Tripoli, Libya; 47 Department of Anesthesiology and Critical Care Medicine, University Hospital of Nancy, Nancy, France; 48 Soins Intensifs, Groupement Hospitalier de L’Ouest Lémanique, Hôpital de Nyon, Nyon, Switzerland

**Keywords:** COVID-19, Pandemic, Intensive care unit, ARDS, Disease dynamics

## Abstract

**Background:**

It remains elusive how the characteristics, the course of disease, the clinical management and the outcomes of critically ill COVID-19 patients admitted to intensive care units (ICU) worldwide have changed over the course of the pandemic.

**Methods:**

Prospective, observational registry constituted by 90 ICUs across 22 countries worldwide including patients with a laboratory-confirmed, critical presentation of COVID-19 requiring advanced organ support. Hierarchical, generalized linear mixed-effect models accounting for hospital and country variability were employed to analyse the continuous evolution of the studied variables over the pandemic.

**Results:**

Four thousand forty-one patients were included from March 2020 to September 2021. Over this period, the age of the admitted patients (62 [95% CI 60–63] years vs 64 [62–66] years, *p* < 0.001) and the severity of organ dysfunction at ICU admission decreased (Sequential Organ Failure Assessment 8.2 [7.6–9.0] vs 5.8 [5.3–6.4], *p* < 0.001) and increased, while more female patients (26 [23–29]% vs 41 [35–48]%, *p* < 0.001) were admitted. The time span between symptom onset and hospitalization as well as ICU admission became longer later in the pandemic (6.7 [6.2–7.2| days vs 9.7 [8.9–10.5] days, *p* < 0.001). The PaO_2_/FiO_2_ at admission was lower (132 [123–141] mmHg vs 101 [91–113] mmHg, *p* < 0.001) but showed faster improvements over the initial 5 days of ICU stay in late 2021 compared to early 2020 (34 [20–48] mmHg vs 70 [41–100] mmHg, *p* = 0.05). The number of patients treated with steroids and tocilizumab increased, while the use of therapeutic anticoagulation presented an inverse U-shaped behaviour over the course of the pandemic. The proportion of patients treated with high-flow oxygen (5 [4–7]% vs 20 [14–29], *p* < 0.001) and non-invasive mechanical ventilation (14 [11–18]% vs 24 [17–33]%, *p* < 0.001) throughout the pandemic increased concomitant to a decrease in invasive mechanical ventilation (82 [76–86]% vs 74 [64–82]%, *p* < 0.001). The ICU mortality (23 [19–26]% vs 17 [12–25]%, *p* < 0.001) and length of stay (14 [13–16] days vs 11 [10–13] days, *p* < 0.001) decreased over 19 months of the pandemic.

**Conclusion:**

Characteristics and disease course of critically ill COVID-19 patients have continuously evolved, concomitant to the clinical management, throughout the pandemic leading to a younger, less severely ill ICU population with distinctly different clinical, pulmonary and inflammatory presentations than at the onset of the pandemic.

**Supplementary Information:**

The online version contains supplementary material available at 10.1186/s13054-022-04065-2.

## Introduction

In March 2020, the coronavirus disease 2019 (COVID-19) pandemic incepted, leading to the largest international health crisis in recent history [1]. Within an impressively short time, the international research community has gained a plethora of new insights regarding the time course of COVID-19 progression and the development of critical illness [2, 3]. Factors prognostic for an unfavourable disease course have been extensively investigated and have provided critical guidance for the pursuit of novel therapeutic approaches [4, 5].

Barely a few months after the pandemic’s outbreak, the first reports describing improvements in mortality in critically ill COVID-19 patients emerged [6–8]. However, such changes can be influenced by a multitude of factors including resource availability, novel therapies and changes in medical practice among others. Moreover, large regional differences have been reported [8–10]. How exactly the characteristics of patients admitted to intensive care units (ICU) and their course of disease have changed, and if such changes can be related to the clinical management and outcome of critically ill COVID-19 patients around the globe, remains elusive.

Evidence of dynamics in the phenotypical expression of COVID-19 critical illness could reveal indications for future preventive and therapeutic strategies and aid in assessing the quality of critical care that is delivered to date. Thus, the present study aimed to answer whether (I) the characteristics of critically ill COVID-19 patients at ICU admission, (II) the temporal course of COVID-19, (III) the clinical management and (IV) ICU outcomes have changed throughout the pandemic.

## Methods

### Study design

On 13 March 2020, the prospective observational *Risk Stratification in COVID-19 patients in the ICU* (RISC-19-ICU) registry was launched to capture COVID-19 features and track characteristics and outcomes of patients with SARS-CoV-2 infections admitted to ICUs [2, 11–14]. The registry (ClinicalTrials.gov Identifier: NCT04357275) has been endorsed by the Swiss Society of Intensive Care Medicine (https://www.sgi-ssmi.ch) and was exempt from the need for additional ethics approval and patient informed consent by the ethics committee of the University of Zurich (KEK 2020-00322). The study complies with the Declaration of Helsinki; the Guidelines on Good Clinical Practice (GCP-Directive) were issued by the European Medicines Agency, the Swiss law and Swiss regulatory authority requirements as well as the regulatory authority requirements in each participating country, and have been designed in accordance with the Strengthening the Reporting of Observational Studies in Epidemiology (STROBE) guidelines for observational studies. The current analysis regarded all patients recorded by 90 centres, in 22 countries, comprising the RISC-19-ICU registry during the period from 1 March 2020 until 30 September 2021 (Additional file 1: e-Appendix 1).

### Inclusion criteria

Inclusion criteria for the RISC-19-ICU registry were (I) a laboratory-confirmed SARS-CoV-2 infection by nucleic acid amplification according to the WHO-issued testing guidelines, and (II) severe manifestation of COVID-19 requiring treatment in an ICU, defined as a hospital ward specialized in the care of critically ill patients with the availability of organ support therapies including invasive mechanical ventilation and non-invasive ventilation.

### Patient data collection

A standardized data set was prospectively collected during the ongoing COVID-19 pandemic for all critically ill COVID-19 patients admitted to the collaborating centres. Data collection was performed through an anonymized electronic case report form managed by the REDCap electronic data capture tool hosted on a secure server by the Swiss Society of Intensive Care Medicine. Data were collected on the day of ICU admission, and on days 1, 2, 3, 5 and 7, including patient characteristics, vital parameters, arterial blood gas analyses and laboratory values, such as inflammatory, coagulation, renal, liver and cardiac markers, treatment modalities and organ support therapies including the use of mechanical ventilation.

### Data transformation

Calculation of the disease severity scores Acute Physiology and Chronic Health Evaluation II (APACHE II), Simplified Acute Physiology Score II (SAPS II) and Sequential Organ Failure Assessment (SOFA) scores as well as the ventilatory ratio [15], a surrogate measure for physiological dead space, was performed using an openly available code library associated with the registry.

In order to allow a discrete numerical summary of the pandemic’s dynamics, apart from modelling time continuously, we sub-divided the pandemic into distinct periods. Three pandemic periods were defined based on the visual analysis of the pandemic’s peaks and SARS-CoV-2 variants in the individual countries composing the registries population (Additional file 1: e-Figure 1–3): Period 1 (1 March 2020 to 30 September 2020), Period 2 (1 October 2020 to 31 January 2021) and Period 3 (1 February 2021 to 30 September 2021). Only variables with a missing rate below 70% were included in the analysis; for a full reporting of missing rates, please refer to Additional file 1: e-Table 1.

**Table 1:** Demographics and baseline characteristics at intensive care unit admission

	Total population	March 2020–September 2020	October 2020–January 2021	February 2021–September 2021
	*n* = 4041	*n* = 1700	*n* = 1543	*n* = 798
Age, years	61 ± 14	62 ± 13	64 ± 14	57 ± 15
Male sex	2753 (70)	1155 (73)	1099 (71)	499 (63)
Body mass index, kg/m^2^	29 ± 6	29 ± 6	29 ± 6	30 ± 6
Time from symptoms to hospital admission, days	9 ± 12	8 ± 7	10 ± 14	10 ± 14
Time from hospital admission to ICU admission, days	3 ± 13	3 ± 9	3 ± 11	3 ± 21
Comorbidities				
Chronic arterial hypertension	1642 (41)	684 (40)	707 (46)	251 (32)
Ischemic heart disease	404 (10)	165 (10)	174 (11)	65 (8)
Chronic heart failure	427 (11)	127 (8)	231 (15)	69 (9)
Diabetes mellitus	989 (25)	366 (22)	435 (28)	188 (24)
Chronic pulmonary disease	414 (10)	191 (11)	169 (11)	54 (7)
Immunosuppression^†^	517 (13)	174 (10)	253 (16)	90 (11)
SOFA Score	8 ± 5	8 ± 5	8 ± 5	6 ± 5
SAPS II Score	36 ± 19	38 ± 19	36 ± 18	32 ± 17
APACHE II Score	16 ± 8	17 ± 9	17 ± 7	14 ± 7
Respiratory support				
Oxygen mask	1333 (33)	663 (39)	671 (43)	428 (46)
High-flow oxygen therapy	464 (13)	101 (7)	220 (16)	143 (23)
Non-invasive mechanical ventilation	372 (11)	166 (11)	137 (10)	69 (11)
Invasive mechanical ventilation	1501 (42)	770 (51)	515 (37)	216 (34)
PaO_2_/FiO_2_ ratio, mmHg	147 ± 115	151 ± 99	148 ± 118	137 ± 144
Ventilatory ratio	2.0 ± 0.9	2.0 ± 0.8	1.9 ± 1.0	2.1 ± 0.9
Vasopressor requirements	681 (17)	272 (16)	285 (18)	124 (16)
Mean arterial pressure, mmHg	85 ± 18	83 ± 15	85 ± 17	90 ± 23
Norepinephrine dose, μg/kg/min	0.1 ± 0.1	0.1 ± 0.2	0.1 ± 0.1	0.1 ± 0.1
White blood cell counts, 10^9^/L	11 ± 6	10 ± 6	11 ± 7	11 ± 6
Neutrophils, 10^9^/L	9 ± 5	8 ± 5	9 ± 5	9 ± 5
Lymphocytes, 10^9^/L	2 ± 3	2 ± 3	2 ± 4	2 ± 3
C-reactive protein, mg/L	143 [78–223]	149 [84–239]	139 [74–219]	134 [73–207]
Procalcitonin, μg/L	0.3 [0.1–0.9]	0.3 [0.2, 1.0]	0.3 [0.1, 0.9]	0.2 [0.1, 0.7]
Interleukin-6, ng/L	91 [32–205]	104 [48–221]	75 [26–211]	83 [29–158]
D-dimers, μg/L	1090 [500–2700]	1169 [600–2805]	1164 [550–2945]	740 [300–1700]
Troponin, ng/L	17 [9–50]	17 [9–46]	22 [10–64]	12 [6–40]
Lactate, mmol/L	1.4 [1.0–2.0]	1.3 [0.9–1.9]	1.4 [1.0–1.9]	1.5 [1.0–2.2]

APACHE II—Acute Physiology And Chronic Health Evaluation; ICU—intensive care unit; PaO_2_/FiO_2_ ratio—partial pressure of arterial O_2_/fraction of inspired O_2_; SOFA—Sequential Organ Failure Assessment; SAPS II—Simplified Acute Physiology Score

Data are presented as mean ± standard deviation, median [interquartile range] or counts (percentages) as appropriated. These are aggregated descriptive data, as opposed to the results of hierarchical, generalized linear mixed-effect modelling as reported in the main results and abstract

^†^Immunosuppression was defined as any of the following: solid organ malignancy, hematologic malignancy, human immunodeficiency virus, hepatitis B or C infection, prescribed immunosuppressive medication

Maximum differences (Δ_late-early_) of vitals and laboratory parameters between ICU admission, day 0 and day 5 of the ICU stay were calculated as follows: $\Delta_{{{\text{late}} - {\text{early}}}} = X \times \left\{ {\max \left( {Y_{Day3} ,Y_{Day5} } \right) - {\text{min}}\left( {Y_{{{\text{Day0}}}} ,Y_{{{\text{Day1}}}} } \right)} \right\} + \left( {1 - X} \right)*\left\{ {\min \left( {Y_{{{\text{Day3}}}} ,Y_{{{\text{Day5}}}} } \right) - \max \left( {Y_{{{\text{Day0}}}} ,Y_{{{\text{Day1}}}} } \right)} \right\}$ where $Y_{Day}$ represents a specific severity score, vital or laboratory parameter at day $\in \left\{ {0, 1, 3, 5} \right\}$ and $X$ is a function such that $$X\left( {Y_{{{\text{Day}}}} } \right) = \left\{ {\begin{array}{*{20}c} 1 & {{\text{if}}\; \left[ {\left( {\frac{{Y_{{{\text{Day3}}}} + Y_{{{\text{Day5}}}} }}{2}} \right) - \left( {\frac{{Y_{{{\text{Day0}}}} + Y_{{{\text{Day1}}}} }}{2}} \right)} \right] > 0} \\ 0 & {{\text{otherwise}}} \\ \end{array} } \right.$$.

### Statistical analysis

Data were modelled by means of hierarchical generalized linear mixed-effect models considering the date of ICU admission as fixed effect while accounting for within-hospital and within-country group nesting, after asserting that the country effects did not introduce extreme effects that could be interpreted as overfitting. The date of ICU admission was modelled as a restricted cubic spline with 3 knots chosen on the 10th, 50th and 90th percentiles and 95% confidence intervals [16]. To analyse the differences in dynamics between ICU survivors and non-survivors, we tested for an interaction term and reported survivor status stratified results. The null hypothesis, formulated as the absence of temporal effect for a specific variable, was assessed by comparing two models (with and without splines) using analysis of deviance with Wald chi-squared tests. Continuous variables were assumed to be Gaussian-distributed, severity scores and time to admission were assumed to be Poisson-distributed, and binary variables to be binomially distributed.

Statistical analysis was performed through a fully scripted data management pathway using the R environment for statistical computing version 4.0.2 [17]. Due to the observational, prospective nature of this cohort study, no power calculations were performed [18]. A two-sided *p* < 0.05 was considered statistically significant. Figures were plotted excluding observations that exceeded the 25th (75th) percentile added to 1.5 times the interquartile range. Values are given as means with standard deviation ± SD, medians with interquartile ranges [IQR] or counts and percentages as appropriate.

## Results

### Overall

From 1 March 2020 until 30 September 2021, date of database closing for this analysis, 4041 critically ill COVID-19 patients were included in 90 centres over 22 countries into the RISC-19-ICU registry. The evolution of SARS-CoV-2 variants in the respective countries is depicted in Additional file 1: e-Figure 3. Similarly, the number of total hospitalized patients as well as patients requiring ICU care in selected countries participating in the registry is displayed in Additional file 1: Figure 4.

Overall, patients were mainly male (70%), aged 61 ± 14 years and presented with a body mass index (BMI) of 29 ± 6 kg/m^2^ (Table 1). They were admitted to the hospital a mean of 9 ± 12 days after symptom onset and had to be referred to the ICU 3 ± 13 days later. At ICU admission, 17% required vasopressor support and 42% were directly intubated and mechanically ventilated. Admission SOFA score amounted to 8 ± 5, and SAPS II and APACHE II scores were 36 ± 19 and 16 ± 8, respectively.

In total, 66% of all patients were invasively mechanically ventilated and 26% of the patients died during their ICU stay.

### Demographics over the time course of the pandemic

One thousand seven hundred (42%) patients were admitted to the respective ICUs between March and September 2020, 1543 (38%) between October 2020 and January 2021, and 798 (20%) between February and September 2021 (Additional file 1: e-Figure 5).

**Fig. 1:**
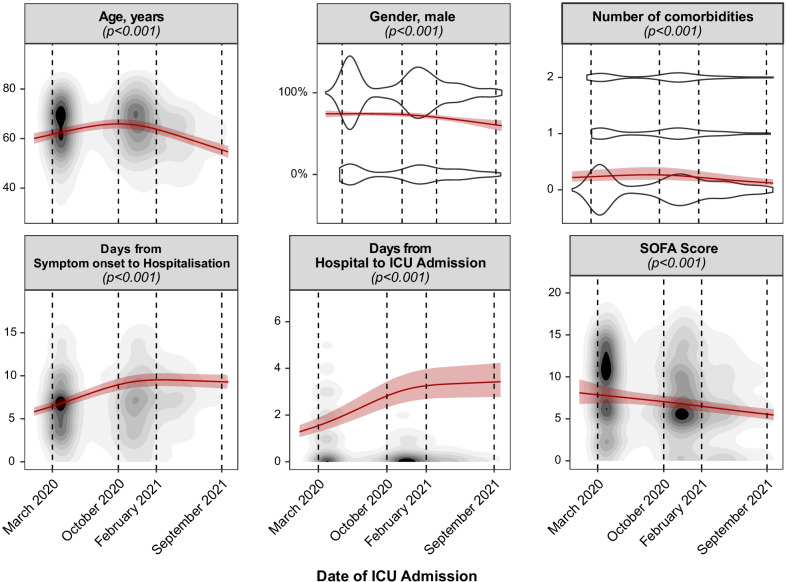
Dynamics of baseline characteristics over the pandemic. Mean effects over the time course of the pandemic, calculated by means of generalized mixed-effect models, are depicted by a red continuous line. 95% confidence intervals of the effect are depicted as shaded red area. The given *p* values originate from an analysis of deviance. Continuous variables are represented by topographic density plots, in which the intensity of the grayscale colouring indicates the highest concentration of values. Categorical variables are represented by violin plots, in which the segmental width of the plot correlates with the concentration of values

Over the time course of the pandemic, severity scores at admission, namely SOFA (March 2020: 8.2 [7.6–9.0], September 2021: 5.8 [5.3–6.4]), age-corrected SAPS II (March 2020: 26 [24–29], September 2021: 19 [17–20]) and APACHE (March 2020: 12.5 [11.5–13.5], September 2021: 8 [7–9]), continuously decreased (*p* < 0.001) (Fig. 1, Additional file 1: e-Figure 6). The percentage of female patients admitted to the ICU started to increase after October 2020 and was highest in September 2021 (March 2020: 26 [23–29]%, October 2020: 26 [24–28]%, February 2021: 30 [28–33]%, September 2021: 41 [35–48]%, *p* < 0.001). On the other hand, while the mean age of patients increased during the first months of the pandemic, it steadily decreased after October 2020 (March 2020: 61 [60–63] years, October 2020: 64 [62–66] years, February 2021: 63 [61–65] years, September 2021: 55 [53–58] years, *p* < 0.001). Similarly, the average number of comorbidities remained constant until October 2020 and then proceeded to decrease until September 2021 (*p* < 0.001). Conversely, the latency between symptom onset and hospitalization (March 2020: 6.7 [6.2–7.2] days, October 2020: 7.7 [7.2–8.3] days, February 2021: 9.5 [8.8–10.3] days, September 2021: 9.7 [8.9–10.5] days, *p* < 0.001) as well as between hospital and ICU admission (March 2020: 1.7 [1.4–2.1] days, October 2020: 2.3 [1.9–2.8] days, February 2021: 3.6 [3.0–4.4] days, September 2021: 4.0 [3.3–5.0] days, *p* < 0.001) steadily increased until February 2021 and then remained constant for the remainder of the studied period.

### Vitals and laboratory findings at ICU admission

**Fig. 2:**
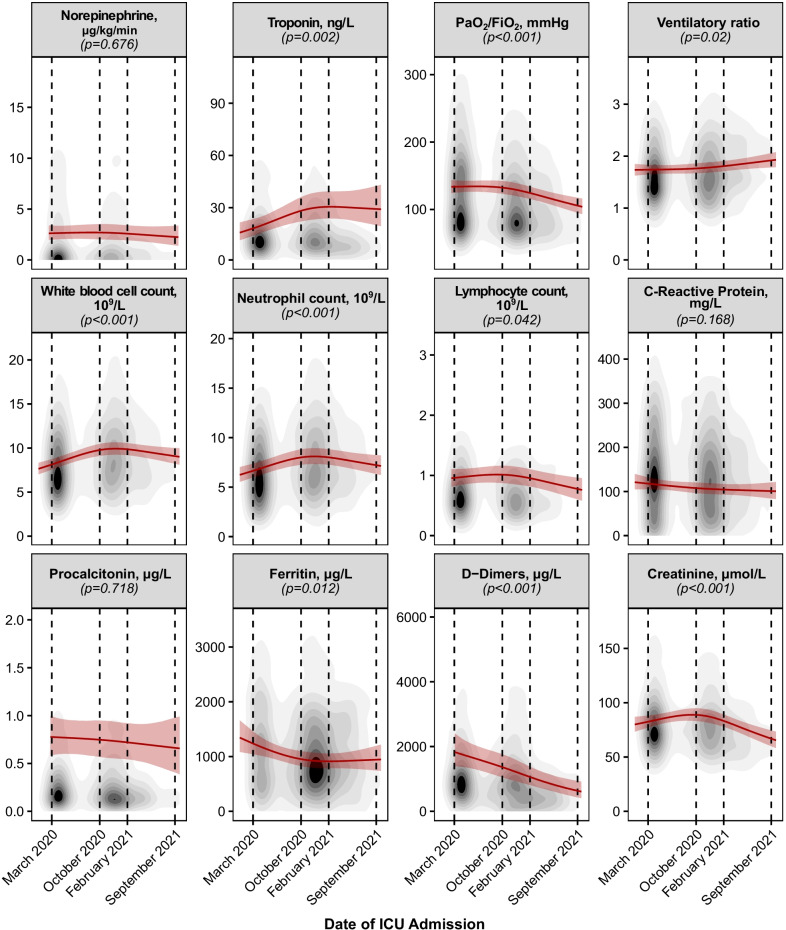
Dynamics of vitals and laboratory parameters at intensive care unit admission. Mean effects over the time course of the pandemic, calculated by means of generalized mixed-effect models, are depicted by a red continuous line. 95% confidence intervals of the effect are depicted as shaded red area. The given *p* values originate from an analysis of deviance. Variables are represented by topographic density plots, in which the intensity of the grayscale colouring indicates the highest concentration of values

Contrasting with the decreasing severity scores, patients admitted to the ICU presented continuously lower paO_2_/FiO_2_ ratios (March 2020: 132 [123–141] mmHg, October 2020: 131 [124–140] mmHg, February 2021: 120 [112–129] mmHg, September 2021: 101 [91–113] mmHg, *p* < 0.001) along with increasing ventilatory ratios (*p* = 0.01); these effects became especially pronounced after October 2020 (Fig. 2, Additional file 1: e-Figure 7). By contrast, D-dimer levels at ICU admission continuously decreased from March 2020 to September 2021 (March 2020: 1722 [1320–2241] μg/l, October 2020: 1581 [1276–1953] mmHg, February 2021: 988 [763–1272] mmHg, September 2021: 506 [338–759] mmHg, *p* < 0.001). Notably, whereas C-reactive protein and procalcitonin levels did remain constant between March 2020 and September 2021, ferritin levels decreased until August 2020 and then remained constant for the remainder of the pandemic. Conversely, leucocyte (March 2020: 8.1 [7.6–8.7] 10^9^/l, October 2020: 8.9 [8.3–9.5] 10^9^/l, February 2021: 9.8 [9.1–10.5] 10^9^/l, September 2021: 9.1 [8.2–10.1] 10^9^/l, *p* < 0.001) and neutrophil counts increased until August 2020 and then continuously decreased for the remainder of the pandemic (*p* < 0.001), whereas lymphocyte counts remained constant until August 2020 and then showed a similar decreasing dynamic as leucocytes and neutrophils (*p* = 0.08).

### Disease progression over the ICU stay

Not only the admission characteristics, but also the dynamics of disease in response to ICU care can change over time. In order to capture these changes in disease progression over the first days of ICU stay, we computed the difference between day 5 and ICU admission and evaluated the change of this parameter (Δ_late-early_) over time.

**Fig. 3:**
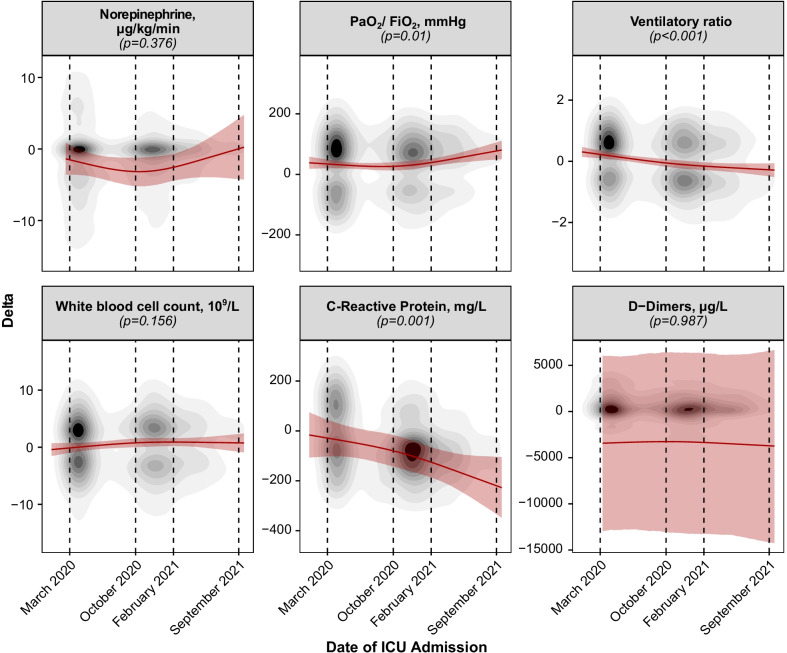
Dynamics of the evolution of vital and laboratory parameters during the first 5 days of intensive care unit stay. To capture the changes in the dynamics of disease over the first days of intensive care unit stay, the difference of a variable between day 5 and day 1 is summarized as parameter (Delta) over time. Mean effects over the time course of the pandemic, calculated by means of generalized mixed-effect models, are depicted by a red continuous line. 95% confidence intervals of the effect are depicted as shaded red area. The given *p* values originate from an analysis of deviance. Variables are represented by topographic density plots, in which the intensity of the grayscale colouring indicates the highest concentration of values

At later stages of the pandemic, the PaO_2_/FiO_2_ ratio increased more pronouncedly during the first 5 days of ICU stay (March 2020: Δ_late-early_ 34 [20–48] mmHg, October 2020: Δ_late-early_ 29 [18–38] mmHg, February 2021: Δ_late-early_ 37 [25–50] mmHg, September 2021: Δ_late-early_ 70 [41–100] mmHg, *p* = 0.05), whereas the ventilatory ratio decreased more markedly (*p* < 0.001) (Fig. 3, Additional file 1: e-Figure 8–9, Additional file 1: e-Table 2). Similarly, C-reactive protein progressively experienced a stronger declining effect over the duration of the pandemic (March 2020: Δ_late-early_ − 29 [− 102 to 44] mg/l, October 2020: Δ_late-early_ − 47 [− 105 to 13] mg/l, February 2021: Δ_late-early_ − 127 [− 189 to − 68] mg/l, September 2021: Δ_late-early_ − 231 [− 352 to − 109] mg/l, *p* = 0.001). However, neither D-Dimer, ferritin and procalcitonin levels, nor leucocyte, neutrophil or lymphocyte count dynamics varied over the time course of the pandemic.

### Medication management

**Fig. 4:**
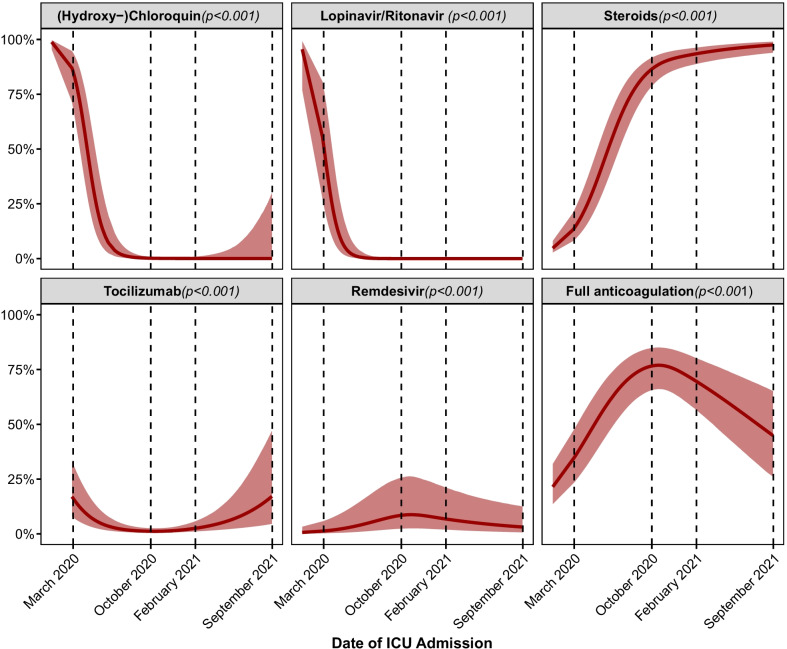
Dynamics of medication management. Mean effects over the time course of the pandemic, calculated by means of generalized mixed-effect models, are depicted by a red continuous line. 95% confidence intervals of the effect are depicted as shaded red area. The given *p* values originate from an analysis of deviance

The use of hydroxychloroquine and ritonavir/lopinavir while widely employed in the first months of the pandemic, dropped to 0% by June 2020 (Fig. 4). Similarly, therapeutic anticoagulation, whereas increasingly employed in the first year of the pandemic, experienced a decline during the second half of the pandemic (March 2020: 35 [24–48]%, October 2020: 77 [66–85]%, February 2021: 70 [57–80]%, September 2021: 45 [26–65]%, *p* < 0.001). Conversely, the use of corticosteroids increased steadily from 14 [9–22] in March 2020, reaching 86 [79–92] by October 2020 and 97 [94–99] by September 2021. Tocilizumab on the other hand was prescribed during the first months of the pandemic, but its prescription saw a halt between June 2020 and February 2021, after which its use steadily increased to 17 [5–47] in September 2021. Most prominently after February 2021, the proportion of vaccinated individuals admitted to the ICU steadily increased (*p* < 0.001) (Additional file 1: e-Figure 10).

### Organ support management and outcomes

**Fig. 5:**
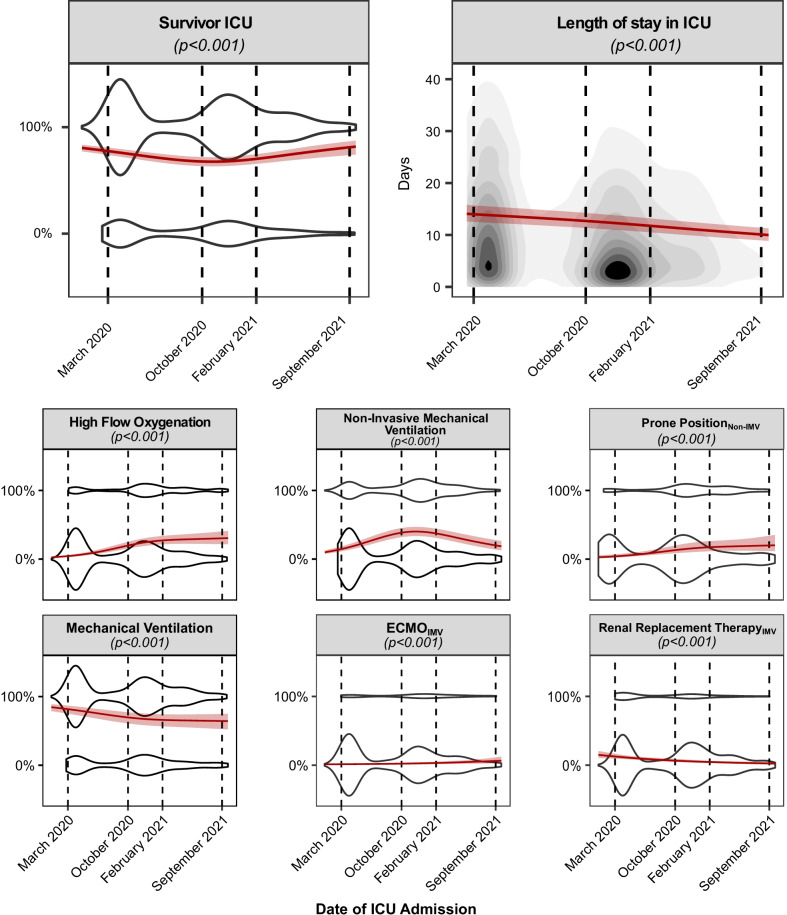
Dynamics of organ support management and outcomes. Mean effects over the time course of the pandemic, calculated by means of generalized mixed-effect models, are depicted by a red continuous line. 95% confidence intervals of the effect are depicted as shaded red area. The given *p* values originate from an analysis of deviance. Continuous variables are represented by topographic density plots, in which the intensity of the grayscale colouring indicates the highest concentration of values. Categorical variables are represented by violin plots, in which the segmental width of the plot correlates with the concentration of values. *IMV* invasive mechanical ventilation

The proportion of patients receiving invasive mechanical ventilation (March 2020: 82 [76–86], October 2020: 76 [70–82]%, February 2021: 70 [61–77]%, September 2021: 74 [64–82]%, *p* < 0.001) and renal replacement therapy decreased (March 2020: 12 [9–16], October 2020: 9 [7–12]%, February 2021: 5 [3–7]%, September 2021: 3 [1–9]%, *p* < 0.001) throughout the pandemic (Fig. 5, Additional file 1: e-Table 3–5). Conversely, more invasively ventilated patients were treated with extracorporeal membrane oxygenation (ECMO) at later stages of the pandemic (March 2020: 0.4 [0.1–1.3] %, October 2020: 0.5 [0.2–2]%, February 2021: 1 [0.4–3]%, September 2021: 3 [1–9]%, *p* < 0.001) (Fig. 4). Overall, more patients were treated with non-invasive mechanical ventilation (March 2020: 14 [11–18], October 2020: 25 [20–31]%, February 2021: 39 [32–46]%, September 2021: 24 [17–33]%, *p* < 0.001) and high-flow oxygen therapy (March 2020: 5 [4–7], October 2020: 10 [8–14]%, February 2021: 24 [19–31]%, September 2021: 20 [14–29]%, v0.001) as the pandemic progressed. Additionally, awake prone position was increasingly employed from February 2021 onwards (March 2020: 50 [43–58]%, October 2020: 47 [40–54]%, February 2021: 45 [38–52]%, September 2021: 52 [42–62]%, *p* < 0.001). Finally, ICU mortality initially worsened until June of 2020 and then progressively improved until September 2021 (March 2020: 23 [19–26], October 2020: 23 [19–26]%, February 2021: 29 [24–33]%, September 2021: 18 [12–24]%, *p* < 0.001), whereas length of ICU stay continuously decreased over the time course of the pandemic (March 2020: 14 [13–16], October 2020: 13 [12–15] days, February 2021: 12 [11–13] days, September 2021: 11 [10–13] days, *p* < 0.001).

### Development of differences between survivors and non-survivors over time

During the pandemic, patients surviving the ICU stay were characterized by a lower age than patients not surviving the ICU, albeit the difference in mean age decreased between March 2020 and October 2020 between both groups (*p* < 0.001) (Additional file 1: e-Figure 11). On the other hand, while non-survivors presented with higher SOFA scores at ICU admission during the whole pandemic, the difference in initial SOFA between non-survivors and survivors grew with the progress of the pandemic (*p* < 0.001).

The PaO_2_/FiO_2_ ratio at admission presented decreasing dynamics in both survivors and non-survivors during the pandemic (*p* < 0.001) (Additional file 1: e-Figure 12). Nevertheless, survivors were characterised by a more pronounced increase in PaO_2_/FiO_2_ ratio (*p* < 0.001) and had a stronger decrease in ventilatory ratio (*p* = 0.03) over the first 5 days of their ICU stay at later stages of the pandemic than non-survivors (Additional file 1: e-Figure 13). On the other hand, C-reactive protein (*p* < 0.001) as well as creatinine levels (*p* < 0.001) albeit lower at admission in survivors than non-survivors in March 2020 progressed to be similar in September 2021 (Additional file 1: e-Figure 12). Strikingly, C-reactive protein dynamics over the first days after ICU admission showed a more pronounced decline in non-survivors after June 2020, as compared to survivors (*p* < 0.001) (Additional file 1: e-Figure 13).

The proportion of survivors not being treated with mechanical ventilation decreased in the first year of the pandemic, to afterwards increase to its initial proportion by September 2021, while this effect was much less pronounced in non-survivors (*p* < 0.001) (Additional file 1: e-Figure 14). Conversely, more survivors than non-survivors were treated with high-flow oxygen over the course of the pandemic (v0.001), whereas a similar proportion of patients received non-invasive mechanical ventilation throughout the pandemic (*p* = 0.40). Interestingly, while survivors presented longer lengths of ICU stay than non-survivors in March 2020, this inverted with non-survivor requiring longer care in the ICU, especially during June 2020 and February 2021, as the pandemic progressed (*p* < 0.001).

## Discussion

This analysis of the RISC-19-ICU registry confirms that the COVID-19 pandemic, especially regarding critically ill patients, has been and remains highly dynamic. Demographics, clinical characteristics, clinical management and outcomes of patients admitted to the ICU have been continuously changing over the last 19 months. Over time, critically ill COVID-19 patients have not only become younger, and increasingly female, but also the time span between symptom onset and ICU admission has become increasingly larger and their degree of overall organ dysfunction at ICU admission has decreased, despite worse initial oxygenation, along with a decrease in ICU mortality.

A plethora of hypotheses might explain these intriguing dynamics. The decrease in age of patients admitted to the ICU is probably a direct effect of the vaccination campaigns in the early 2021, which initially targeted mainly the elderly population [19]. However, more complex social aspects, such as the increasing non-compliance with public health measures of young adults, but not of elderly individuals, after extensive lockdowns and the high initial death toll on the most fragile elderly population, should also be considered [20]. Additionally, whilst the largely experimental antiviral and anti-inflammatory therapies employed in the early months of the pandemic failed to prove their efficacy [21, 22], the progressive use of dexamethasone after the publication of the RECOVERY landmark trial in June 2020 [4] as well as remdesivir in November 2020 [23] and tocilizumab in February 2021 [24] could be reasons for the increasing ICU survival in subsequent COVID-19 waves [25]. Indeed, the more pronounced decrease in C-reactive protein over the ICU stay and the concomitant increase in leucocyte and specifically neutrophil counts at later stages of the pandemic may seem to reflect the systematic initiation of corticosteroids, possibly conjointly employed with tocilizumab [4, 26].

The younger age of patients admitted to the ICU could be the primary factor accounting for the prolongation of periods between symptom onset and hospital admission as well as hospital admission and ICU admission. Younger patients, presenting with fewer comorbidities, have greater pulmonary reserves and can compensate lower respiratory tract infections more effectively than elders [27, 28]. Additionally, increasing experience in the treatment of acute hypoxemic respiratory failure due to COVID-19 in hospital wards including more advanced non-invasive respiratory support strategies and the progressive use of awake prone positioning might have postponed or completely avoided ICU admission [8, 29, 30]. This time lag could also explain the more pronounced hypoxemia and higher dead space at ICU admission in the later months of the pandemic possibly induced by patient self-inflicted lung injury (P-SILI) and contrasting with the lower overall severity scores [12, 31]. Furthermore, as previously mentioned, early initiation of dexamethasone and remdesivir, but also the increasing use of intermediate and therapeutic anticoagulation after the first wave might be responsible for the delayed ICU admission [4, 5, 23]. The widespread implementation of intermediate and therapeutic anticoagulation strategies already in wards and intermediate care units at later stages of the pandemic could also explain the decrease in D-dimer levels at ICU admission. On the other hand, the lack of change in D-dimer dynamics over the first 5 days of the ICU stay could reflect the demonstrated lack of efficacy of these therapies when patients have already reached the ICU setting [32].

Similar to other reports, the use of invasive mechanical ventilation in the ICU has declined over the course of the pandemic, this seems to be primarily the effect of the increased use of high-flow oxygen therapy and non-invasive ventilation strategies, synergistically used with awake prone position, albeit the younger age and reduced organ dysfunction could also be plausible reasons [6–8]. However, the use of ECMO as salvage therapy in invasively mechanically ventilated patients substantially increased during the pandemic. Whether the latter reflects increased lung damage in ultimately intubated patients [12] or less strict regulations for the initiation of ECMO as a consequence of the patient’s younger age [33], remains unknown. The decreasing length of stay and increasing survival in ICUs worldwide, albeit possibly confounded by age and lower disease severity, are, however, reassuring and evidence of the immense advances made in the care of critically ill COVID-19 patients.

Our study has many strengths. Its multicentre design including 90 centres in 22 countries make the observations generalizable to a wide international critically ill COVID-19 population. Its prospective design allows for a near real-time analysis of the characteristics of critically ill COVID-19 patients throughout the pandemic. This allows insights not only into the epidemiological changes of the COVID-19 pandemic, but also capturing effects of factors that might affect the course of the pandemic, such as public health policies, the prevalence of novel virus variants, the introduction of new treatments and the increasing rates of vaccination. Moreover, using a unique statistical approach (Δ_late-early_) this study was able to capture subtle changes in the dynamics of disease course during the first days after ICU admission.

The present analysis, however, also has important limitations. *First*, the high intra- and inter-country heterogeneity regarding socio-economic differences (Additional file 1: e-Figure 15), delivery of care, ICU admission regulations, staff-to-patient ratios and temporal incidence of critically ill patients can limit the interpretability of the data. Additionally, temporal changes in ICU admission policies and an increasing use of advanced respiratory support and specialized medication in non-ICU hospital wards, including high-flow oxygenation and non-invasive mechanical ventilation, could bias the epidemiology of patients admitted to the ICU. We, however, employed centre-within-country nesting to minimize the bias induced by these heterogeneous effects. *Second*, although standard clinical reporting form and codebook were available, a substantial fraction of fields reported missing data, possibly limiting the interpretation of the results. Nevertheless, by only considering variables with a missing rate below 70% and through rigorous monitoring of data quality the effect of missingness could be reasonably mitigated. *Finally*, some centres that included patients during the first wave did not recruit patients during subsequent waves. However, the larger most representative centres did continuously include all their patients throughout the 19 months of the pandemic. This, in combination with within centre random effects, allowed to reduce the residual bias originating from inconsistent recruitment strategies.

## Conclusions

In conclusion, this study confirms the continuous evolution of the COVID-19 pandemic and provides important insights into the epidemiological dynamics of critically ill patients admitted to ICUs worldwide, as well as to their clinical management. During subsequent waves of the pandemic, patients admitted to the ICU were younger, had less comorbidities, were less severely ill, had an increased survival and a shorter length of stay. Concomitantly, a higher proportion of patients were treated with corticosteroids as well as tocilizumab and less patients were mechanically ventilated. Close monitoring of the changing characteristics and disease course of critically ill COVID-19 patients is essential to continuously guide public health policies, prospectively tailor new therapeutical approaches and promote evidence-based management during this health crisis.

## Supplementary Information

**Additional file 1.** Online Supplementary Material.

## Data Availability

Any intensive care unit or other centre treating critically ill COVID-19 patients is invited to join the RISC-19-ICU registry at https://www.risc-19-icu.net. Analyses on the full data set may be requested by all collaborating centres after approval of the study protocol by the registry board.

## Code availability

Code for registry-specific data transformation and statistical analysis has been made available for collaborative development on the GitHub and Zenodo repositories. The registry protocol and data dictionary may be found at https://www.risc-19-icu.net.
