# Supplementary material for: Dynamics of disease characteristics and clinical management of critically ill COVID-19 patients over the time course of the pandemic: an analysis of the prospective, international, multicentre RISC-19-ICU registry
Source: Crit Care. 2022 Jul 4;26:199. doi: 10.1186/s13054-022-04065-2 (PMC9254551; doi:10.1186/s13054-022-04065-2)

Online Supplementary Material

Dynamics of disease characteristics and clinical management of critically ill COVID-19 patients over the time course of the pandemic: An analysis of the prospective, international, multicentre RISC-19-ICU registry

Wendel Garcia PD, Moser A, Jeitziner MM, Aguirre-Bermeo H, Arias-Sanchez P, Apolo J, Roche-Campo F, Franch-Llasat D, Kleger DR, Schrag C, Pietsch U, Filipovic M, David S, Stahl K, Bouaoud S, Ouyahia A, Fodor P, Locher P, Siegemund M, Zellweger N, Cereghetti S, Schott P, Gangitano G, Wu MA, Alfaro-Farias M, Vizmanos-Lamotte G, Ksouri H, Gehring N, Rezoagli E, Turrini F, Lozano-Gómez H, Carsetti A, Rodríguez-García R, Yuen B, Baltussen Weber A, Castro P, Escos-Orta JO, Dullenkopf A, Martín-Delgado MC, Aslanidis T, Perez HM, Hillgaertner F, Ceruti S, Franchitti Laurent M, Marrel J, Colombo R, Laube M, Fogagnolo A, Studhalter M, Wengenmayer T, Gamberini E, Buerkle C, Buehler PK, Keiser S, Elhadi M, Montomoli J, Guerci P, Fumeaux T, Schuepbach RA, Jakob SM, Que YA, Hilty MP

On behalf of the RISC-19-ICU Investigators

e-Appendix 1: The RISC-19-ICU Investigators 3
e-Figure 1: Daily new SARS-COV-2 positive cases in the countries participating in the registry 8
e-Figure 2: Daily new SARS-COV-2 positive cases in the countries participating in the registry (aggregated for all countries)....................9
e-Table 1: Missing Values....................10
e-Figure 3: Daily distribution of SARS-CoV-2 variants in the countries participating in the registry....................11
e-Figure 4: Daily hospitalizations and intensive care unit admissions in a selected sample of participating countries....................12
e-Figure 5: Study flow chart 13
e-Figure 6: Dynamics of baseline characteristics over the pandemic *(extended)* 14
e-Figure 7: Dynamics of vitals and laboratory parameters at intensive care unit admission *(extended)*....................15
e-Figure 8: Dynamics of the evolution of vital and laboratory parameters during the first five days of intensive care unit stay *(extended 1)* 16
e-Figure 9: Dynamics of the evolution of vital and laboratory parameters during the first five days of intensive care unit stay *(extended 2)* 17
e-Figure 10: Evolution of the proportion of vaccinated patients admitted to the intensive care unit....................18
e-Table 2: Dynamics of $\Delta_{\text{late-early}}$, representing the difference between day 5 and intensive care unit admission, throughout the pandemic 19
e-Table 3: Respiratory and organ support during intensive care unit stay and outcomes *(whole population)* 20
e-Table 4: Respiratory and organ support during intensive care unit stay and outcomes *(invasive mechanically ventilated)* 21
e-Table 5: Respiratory and organ support during intensive care unit stay and outcomes *(non-invasive mechanically ventilated)*....................22
e-Figure 11: Dynamics of baseline characteristics over the pandemic *(stratified by intensive care unit survival)*....................23
e-Figure 12: Dynamics of vitals and laboratory parameters at intensive care unit admission *(stratified by intensive care unit survival)* 24
e-Figure 13: Dynamics of the evolution of vital and laboratory parameters during the first five days of intensive care unit stay *(stratified by intensive care unit survival)*....................25
e-Figure 14: Dynamics of outcomes and organ support strategies *(stratified by intensive care unit survival)* 26
e-Figure 15: Gross domestic product per capita for selected countries participating in the registry 2020....................27

<u>e-Appendix 1:</u> The RISC-19-ICU Investigators

Registry Board

Matthias P. Hilty†, Pedro D. Wendel Garcia†, Reto A. Schuepbach, Jonathan Montomoli, Philippe Guerci, Thierry Fumeaux

†*initiated the registry and designed the e-CRF*

Local Center Collaborators

Listed in alphabetical order of countries and cities

Algeria
Centre Hospitalo Universitaire - Saadna Mohamed Abdnour, Setif: Souad Bouaoud, Amel Ouyahia, Meriem Abdoun, Mounira Rais.

Andorra
Hospital Nostra Senyora de Meritxell, Escaldes-Engordany: Mario Alfaro-Farias, Gerardo Vizmanos-Lamotte, Angel Caballero.

Austria
Kepler University Hospital GmbH and Johannes Kepler University, Linz: Thomas Tschoellitsch, Jens Meier.

Ecuador
Hospital Vicente Corral Moscoso, Cuenca: Hernán Aguirre-Bermeo, Pedro Arias-Sanchez, Janina Apolo, Luis A. Martinez, Hugo Tirapé-Castro.

Egypt
Aswan University Hospital, Aswan: Islam Galal.
Mansoura University Hospitals, Mansoura: Samar Tharwat.
Zagazig University Hospitals, Zagazig: Ibrahim Abdehaleem.

France
Clinique Louis Pasteur, Essey-lès-Nancy: Geoffrey Jurkolow.

University Hospital of Nancy, Nancy: Philippe Guerci, Emmanuel Novy, Marie-Reine Losser.

Germany
Medical Center University of Freiburg, Freiburg: Tobias Wengenmayer, Viviane Zotzmann.
Medical School Hannover, Hannover: Sascha David, Klaus Stahl, Benjamin Seeliger, Tobias Welte.

Greece
St. Paul General Hospital of Thessaloniki, Thessaloniki: Theodoros Aslanidis.

Hungary
University of Szeged, Szeged: Anita Korsos.

Iraq
Al-Hussaien Teaching Hospital, Samawa: Luqman Abdulkhudhur Ahmed.
Al-Nassiryah Teaching Hospital, Thi Qar: Hashim Talib Hashim.

Iran
Shiraz University of Medical Sciences, Shiraz: Reza Nikandish.

Italy
Azienda Ospedaliero Universitaria Ospedali Riuniti di Ancona, Ancona: Andrea Carsetti, Erika Casarotta, Paolo Giaccaglia.
Policlinico San Marco - Gruppo Ospedaliero San Donato, Bergamo: Emanuele Rezoagli, Matteo Giacomini, Aurora Magliocca.
Ospedale Bufalini, Cesena: Giuliano Bolondi.
Azienda Unità Sanitaria Locale della Romagna, Faenza: Antonella Potalivo
Azienda Ospedaliero-Universitaria di Ferrara, Cona: Alberto Fogagnolo.
IRCCS Centro Cardiologico Monzino, Milan: Luca Salvi.
ASST Fatebenefratelli Sacco - "Luigi Sacco" Hospital, Milan: Maddalena A. Wu, Chiara Cogliati, Riccardo Colombo, Emanuele Catena.
Azienda Ospedaliera Universitaria di Modena, Modena: Fabrizio Turrini, Maria S. Simonini, Silvia Fabbri.
Ospedale Infermi, Rimini: Jonathan Montomoli, Emiliano Gamberini, Gianfilippo Gangitano, Maria M. Bitondo, Francesca Maciopinto, Enrico de Camillis, Marta Venturi.

Fondazione Policlinico Universitario A. Gemelli IRCCS, Rome: Maria Grazia Bocci, Massimo Antonelli.

Libya
Ghadames General Hospital, Ghadames: Arowa Alansari.
Almwasfat Clinic, Tripoli: Abdurraouf Abusalama.
Alzintan Hospital, Tripoli: Osama Omar
Tripoli Central Hospital, Tripoli: Muhannud Binnawara
Tripoli Medical Center, Tripoli: Hind Alameen
University of Tripoli, Tripoli: Muhammed Elhadi
Sorman Teaching Hospital, Sorman: Abdulmueti Alhadi
Tobruk Medical Centre, Tobruk: Ahmed Arhaym

Netherlands
Erasmus Medical Center, Rotterdam: Diederik Gommers, Can Ince.

Palestine
Nasser Medical Complex, Gaza: Mustafa Jayyab
Al-Ahli Hospital, Hebron: Mohammed Alsharif.

Spain
Complejo Hospitalario Universitario A Coruña, A Coruña: Raquel Rodríguez-García, Jorge Gámez-Zapata, Xiana Taboada-Fraga.
Hospital Clínic de Barcelona, Barcelona: Pedro Castro, Javier Fernandez, Enric Reverter.
Hospital General San Jorge, Huesca: Arantxa Lander-Azcona, Jesús Escós-Orta.
Hospital Universitario de Torrejon, Madrid: Maria C. Martín-Delgado, Angela Algaba-Calderon.
Hospital Verge de la Cinta de Tortosa, Tarragona: Ferran Roche-Campo, Diego Franch-Llasat, Pablo Concha, Esther Sauras-Colón.
Hospital Clínico Universitario Lozano Blesa, Zaragoza: Herminia Lozano-Gómez, Begoña Zalba-Etayo, Maria P. Montes.

Switzerland
Kantonsspital Aarau, Aarau: Marc P. Michot, Alexander Klarer, Rolf Ensner.
Zuger Kantonsspital AG, Baar: Peter Schott, Severin Urech.
Universitaetsspital Basel, Basel: Martin Siegemund, Nuria Zellweger, Caroline E. Gebhard, Alexa Hollinger.

St. Claraspital, Basel: Lukas Merki, Adriana Lambert.
Spitalzentrum Biel, Biel: Marcus Laube
University Hospital Bern, Bern: Marie M. Jeitziner, Andre Moser, Yok-Ai Que, Stephan M. Jakob.
Lindenhofspital, Bern: Jan Wiegand.
Spital Buelach, Buelach: Bernd Yuen, Barbara Lienhardt-Nobbe, Andrea Westphalen.
Regionalspital Emmental AG, Burgdorf: Petra Salomon.
Kantonsspital Graubuenden, Chur: Frank Hillgaertner, Marianne Sieber.
Spital Thurgau, Frauenfeld: Alexander Dullenkopf, Giulio Barana.
Hopital cantonal de Fribourg, Fribourg: Hatem Ksouri, Govind O. Sridharan.
University Hospitals of Geneva, Geneva: Sara Cereghetti, Filippo Boroli, Jerome Pugin, Serge Grazioli.
Spital Grabs, Grabs: Christian Bürkle.
See-Spital Horgen & Kilchberg, Horgen: Julien Marrel, Mirko Brenni.
Hirslanden Clinique Cecil, Lausanne: Isabelle Fleisch.
University Hospital Lausanne, Lausanne: Marie-Helene Perez, Anne-Sylvie Ramelet.
Kantonsspital Baselland, Liestal: Anja Baltussen Weber, Peter Gerecke, Andreas Christ.
Clinica Luganese Moncucco, Lugano: Samuele Ceruti, Andrea Glotta, Maira Biggiogero.
Spital Maennedorf AG, Maennedorf: Katharina Marquardt.
Spital Thurgau, Muensterlingen: Tobias Hübner, Thomas Neff.
Schweizer Paraplegikerzentrum Nottwil, Nottwil: Hermann Redecker.
Groupement Hospitalier de l'Ouest Lémanique - Hôpital de Nyon, Nyon: Thierry Fumeaux, Mallory Moret-Bochatay, Marco Betello.
Spitalzentrum Oberwallis, Visp: Friederike Meyer zu Bentrup
Kantonsspital Olten, Olten: Michael Studhalter.
Spital Oberengadin, Samedan: Michael Stephan.
Kantonsspital Schaffhausen, Schaffhausen: Nadine Gehring.
Spital Schwyz, Schwyz: Daniela Selz.
Kantonsspital St. Gallen, St. Gallen: Gian-Reto Kleger, Claudia Schrag, Urs Pietsch, Miodrag Filipovic.
Kantonsspital Nidwalden, Stans: Anette Ristic.
Spital Simmental-Thun-Saanenland AG, Thun: Antje Heise.

Établissements hospitaliers du Nord vaudois, Yverdon- les-Bains: Marilene Franchitti Laurent, Jean-Christophe Laurent.
Hirslanden Klinik Im Park, Zurich: Tomislav Gaspert.
Klinik Hirslanden, Zurich: Christoph Haberthuer.
Stadtspital Triemli, Zurich: Patricia Fodor, Pascal Locher
University Hospital Zurich, Zurich: Pedro D. Wendel Garcia, Matthias P. Hilty, Reto Schuepbach, Stefanie Keiser, Dorothea Heuberger, Jan Bartussek, Philipp Bühler, Silvio Brugger, Eva-Maria Kleinert, Kim-Jana Fehlbier.

Syria
Aleppo University Hospital, Aleppo: Aghyad Danial.
Hamer National Hospital, Hama: Maher Almousa.
National Hospital of Lattakia, Lattakia: Yazan Abdulbaki.

Turkey
Ondokuz Mayis University Hospital, Samsun: Kamil Sannah.
Samsun Training and Research Hospital, Samsun: Elif Colak.

United Kingdom
Royal Brompton and Harefield NHS, London: Nandor Marczin

Yemen
Al-Yemen Alseed Hospital, Sanaa: Saba Al-Ameri

e-Figure 1: **Daily new SARS-COV-2 positive cases in the countries participating in the registry**

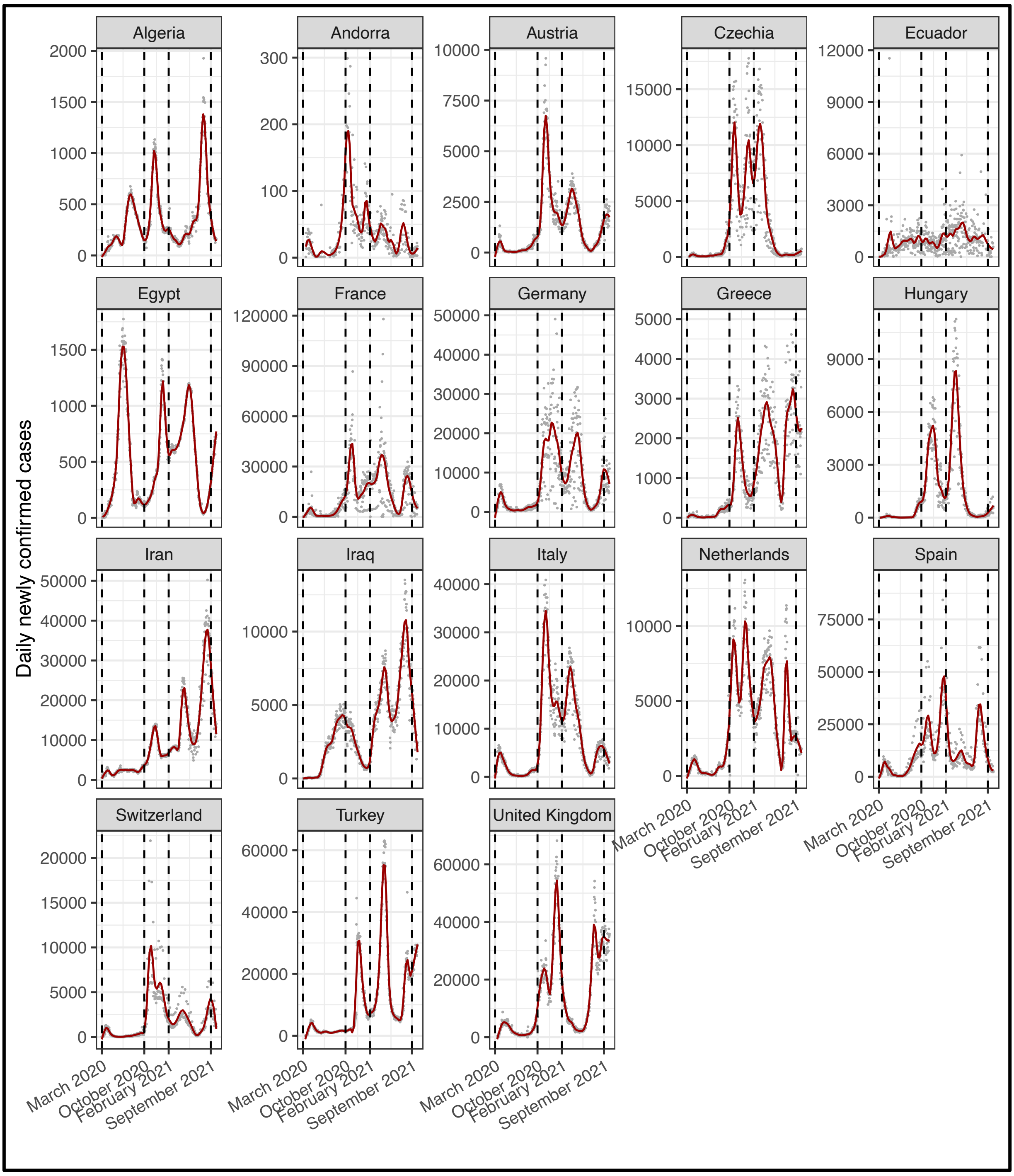

Gray dots indicate daily new cases (>0). The red line represents the fit from a locally estimated scatterplot smoother. Data source: COVID−19 Data Repository by the Centre for Systems Science and Engineering (CSSE) at Johns Hopkins University; https://github.com/CSSEGISandData/COVID−19 (accessed Oct 15, 2021).

<u>e-Figure 2:</u> Daily new SARS-COV-2 positive cases in the countries participating in the registry (aggregated for all countries)

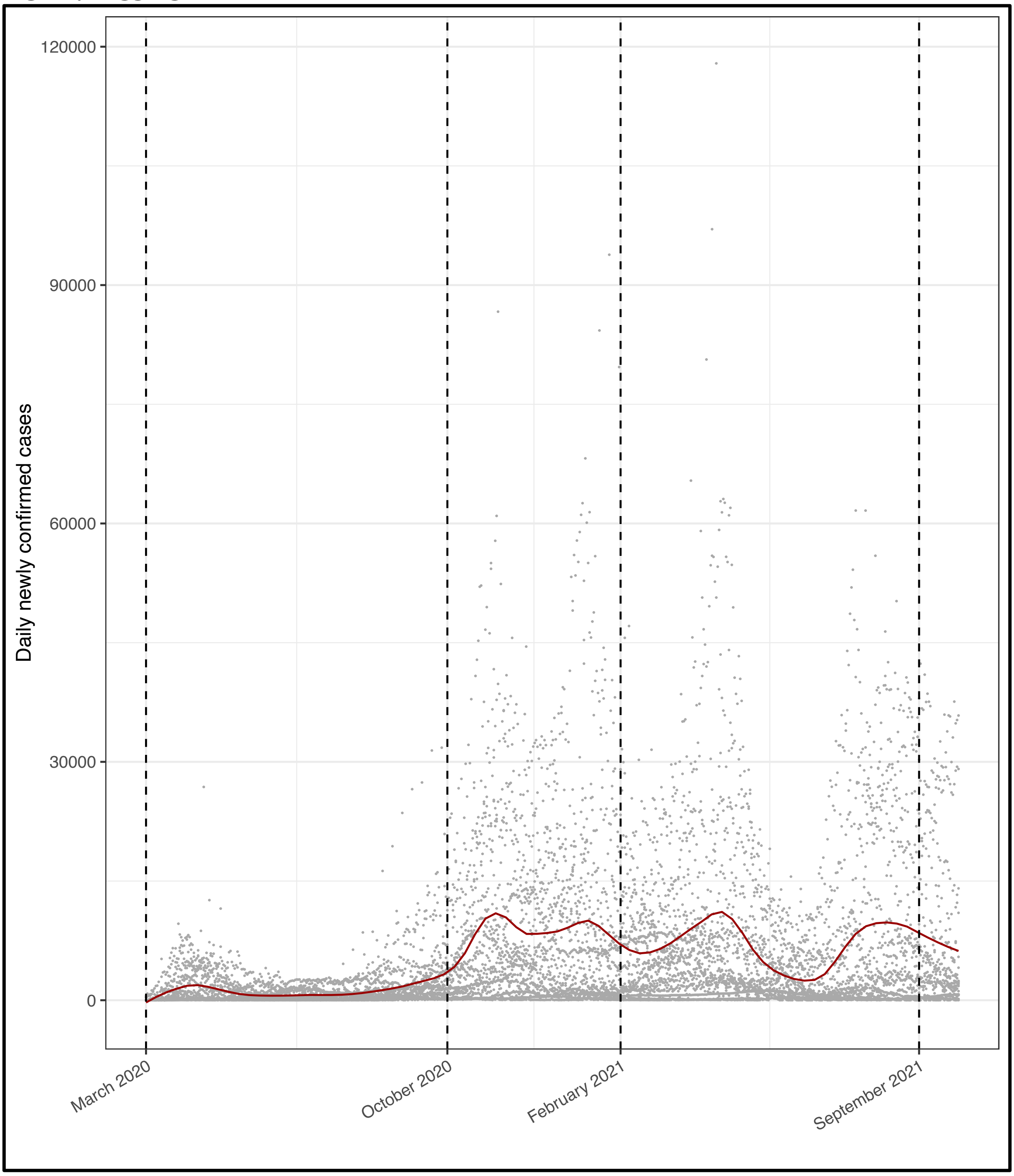

Gray dots indicate daily new cases (>0). The red line represents the fit from a locally estimated scatterplot smoother. <u>Data source:</u> COVID−19 Data Repository by the Centre for Systems Science and Engineering (CSSE) at Johns Hopkins University; https://github.com/CSSEGISandData/COVID−19 (accessed Oct 15, 2021).

e-Table 1: Missing Values

Variable	Missing Values
Age	109 (2.7%)
Gender	114 (2.8%)
Body Mass Index	510 (12.6%)
Mechanical ventilation	501 (12.4%)
Days from hospitalisation to Intensive Care Unit	443 (11.0%)
Days from symptoms to hospitalisation	495 (12.2%)
Mean arterial pressure	912 (22.6%)
CK	2570 (63.6%)
Troponin	2552 (63.2%)
PaO_2/FiO_2 ratio	1177 (29.1%)
Ventilatory ratio	1028 (25.4%)
Temperature	599 (14.8%)
White blood cell count	810 (20.0%)
Neutrophil count	1390 (34.4%)
Lymphocyte count	1360 (33.7%)
C-reactive protein	1072 (26.5%)
Procalcitonin	1894 (46.9%)
Ferritin	2537 (62.8%)
D-Dimers	1940 (48.0%)
Lactate dehydrogenase	2155 (53.3%)
Haematocrit	813 (20.1%)
Bilirubin	1696 (42.0%)
Creatinine	892 (22.1%)
Urea	1486 (36.8%)
Sodium	344 (8.5%)
Potassium	333 (8.2%)
Arterial pH	694 (17.2%)
Glasgow coma scale	622 (15.4%)
Survivor ICU	496 (12.3%)
Length of stay in ICU	753 (18.6%)

<u>e-Figure 3:</u> Daily distribution of SARS-CoV-2 variants in the countries participating in the registry

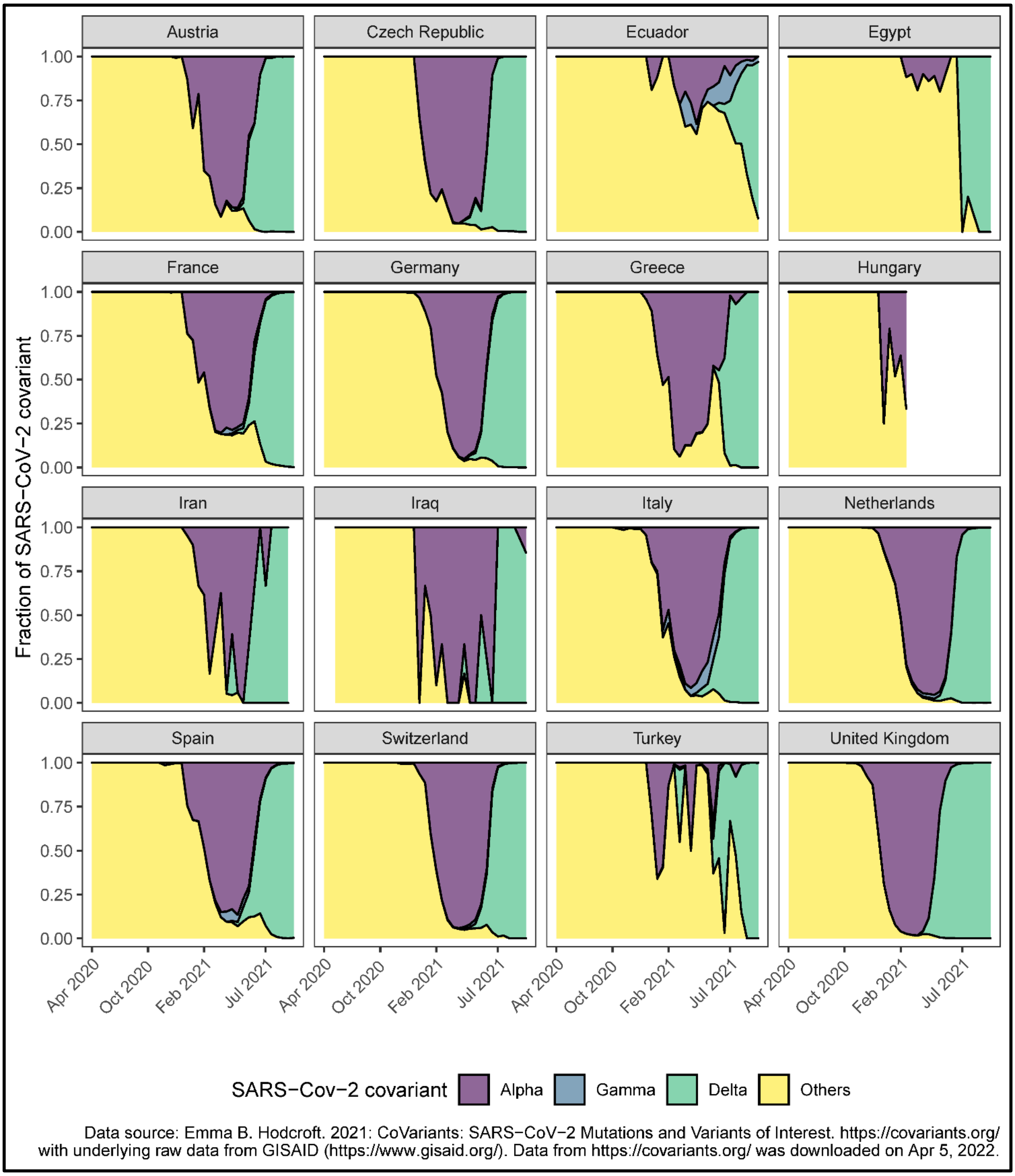

e-Figure 4: **Daily hospitalizations and intensive care unit admissions in a selected sample of participating countries**

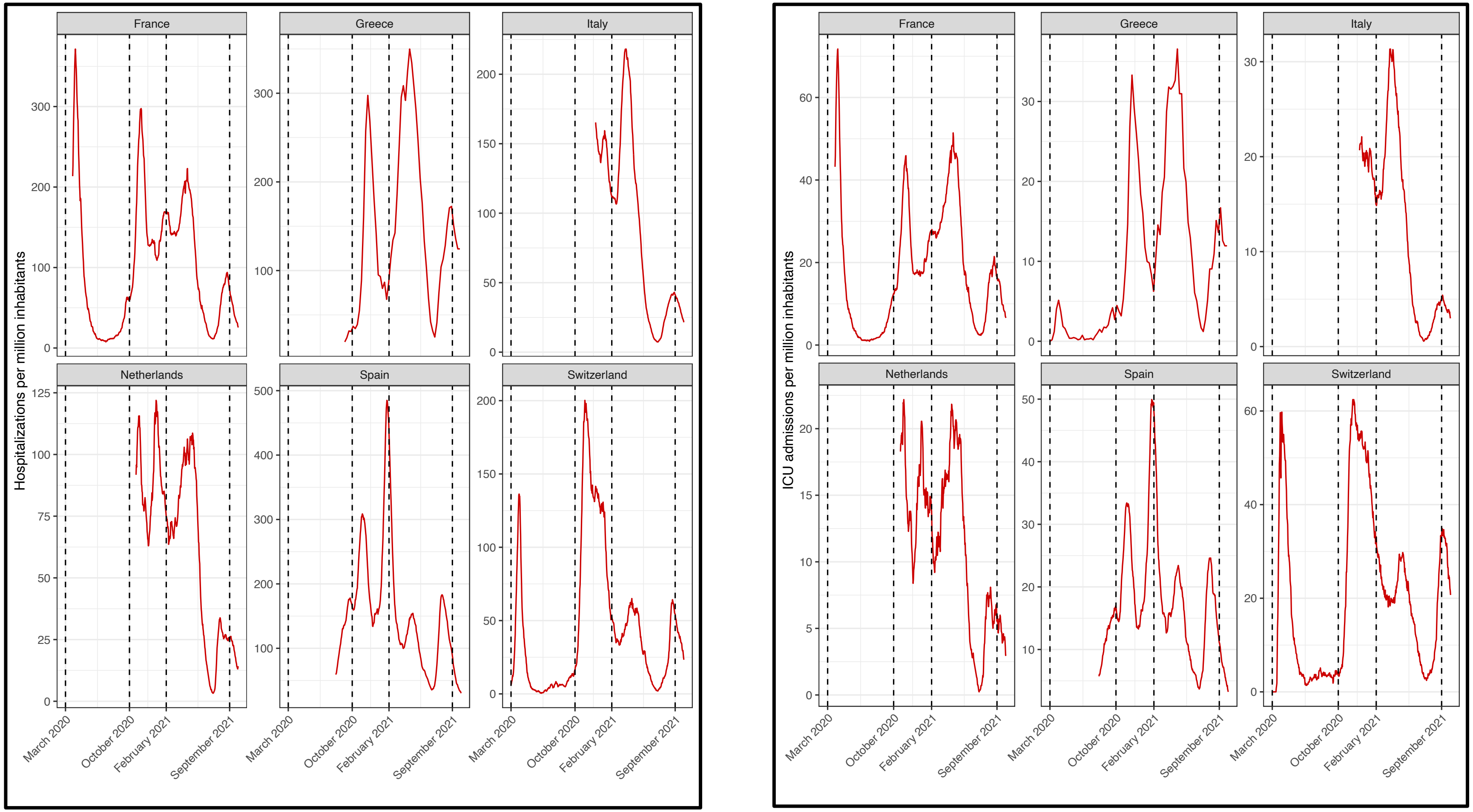

Red line represents the per-country daily hospitalizations and intensive care unit admissions. Data source: Hannah Ritchie et al. (2020) - "Coronavirus Pandemic (COVID-19)". Published online at OurWorldInData.org. Retrieved from: 'https://ourworldindata.org/coronavirus' downloaded on Apr 25, 2022.

e-Figure 5: **Study flow chart**

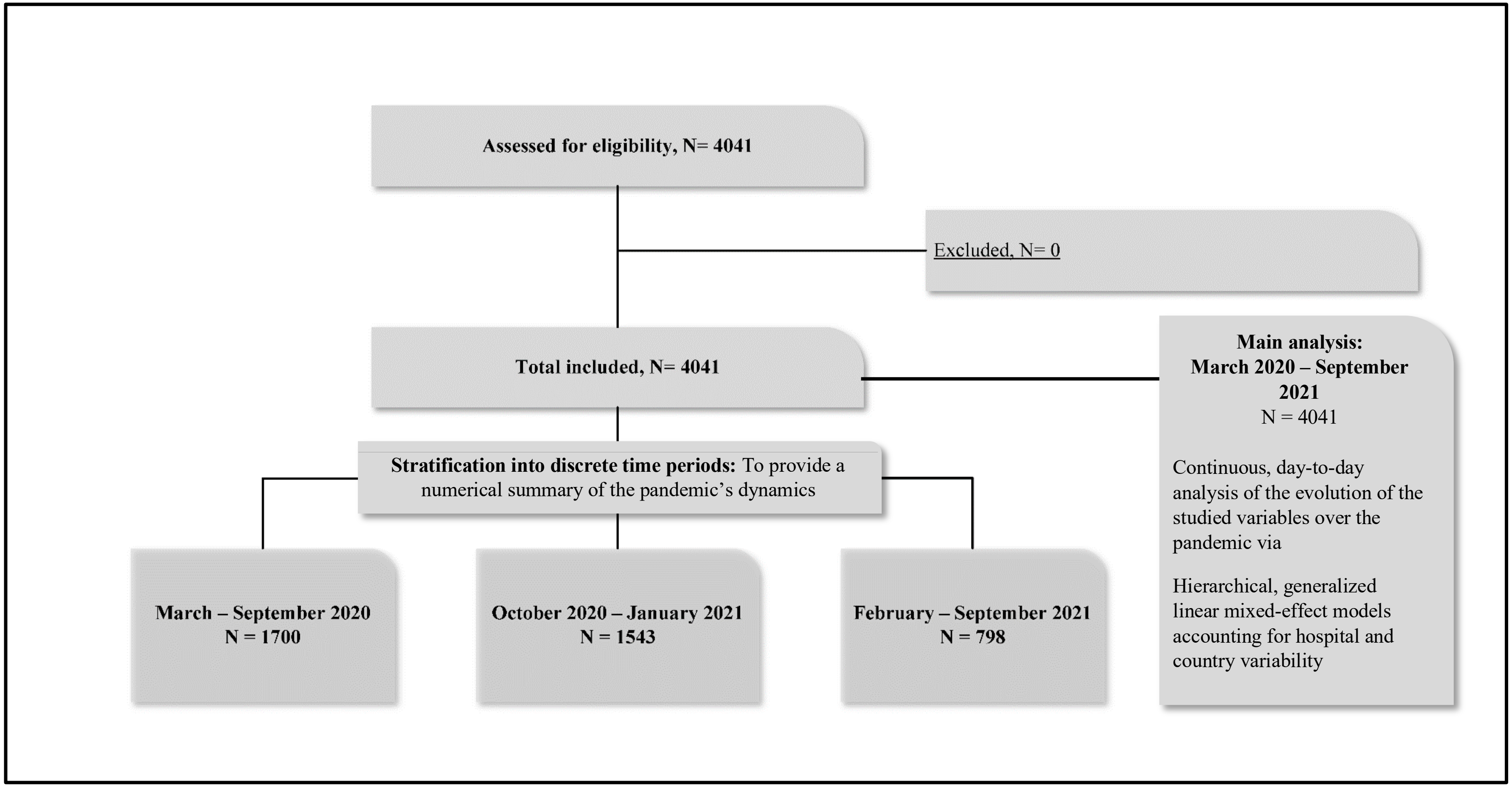

e-Figure 6: Dynamics of baseline characteristics over the pandemic *(extended)*

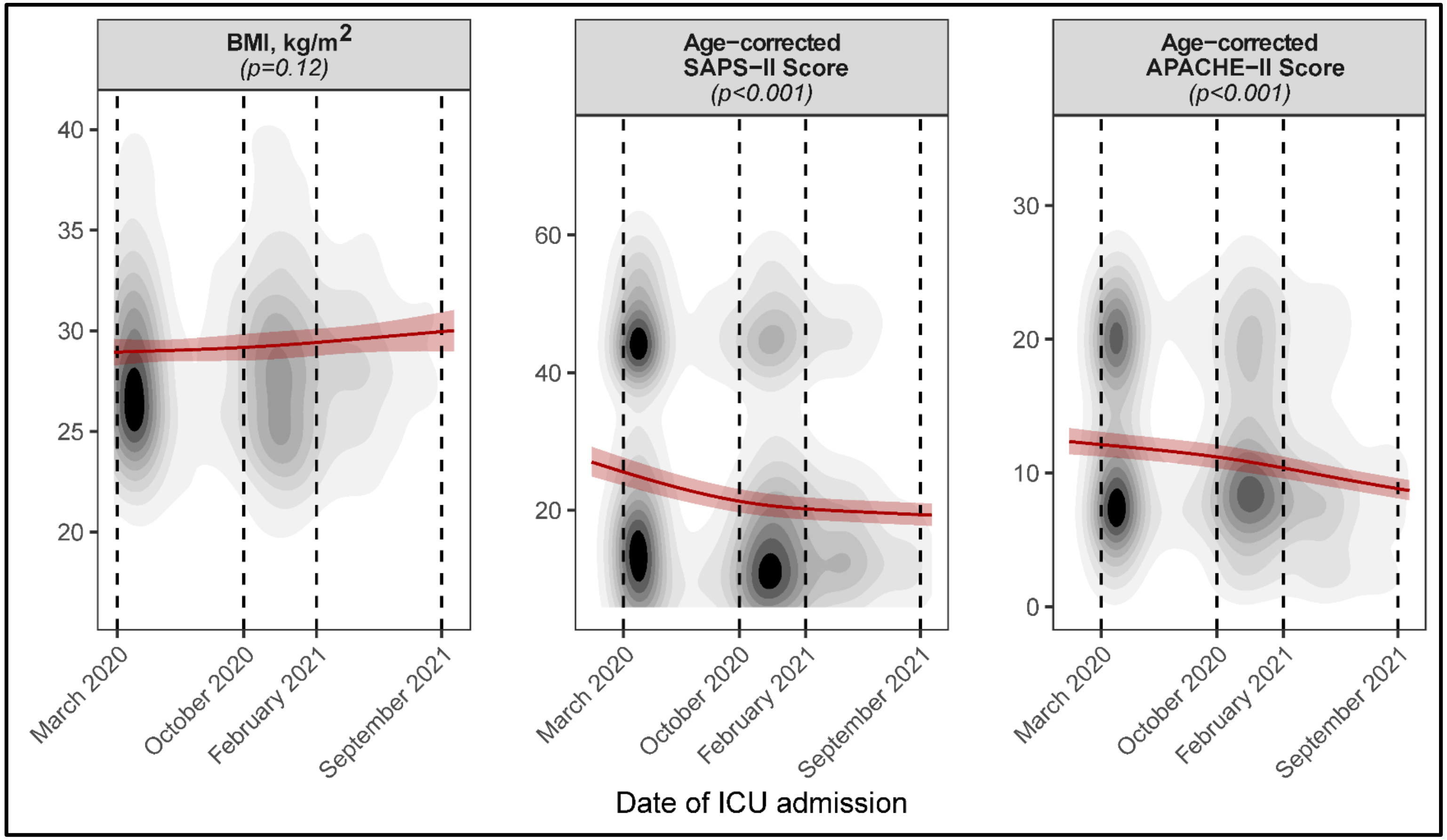

Mean effects over the time course of the pandemic, calculated by means of generalized mixed effect models, are depicted by a red continuous line. 95% confidence intervals of the effect are depicted as shaded red area. The given p-values originate from an analysis of deviance. Continuous variables are represented by topographic density plots, in which the intensity of the grayscale coloring indicates the highest concentration of values. Categorical variables are represented by violin plots, in which the segmental width of the plot correlates with the concentration of values.

<u>e-Figure 7:</u> Dynamics of vitals and laboratory parameters at intensive care unit admission *(extended)*

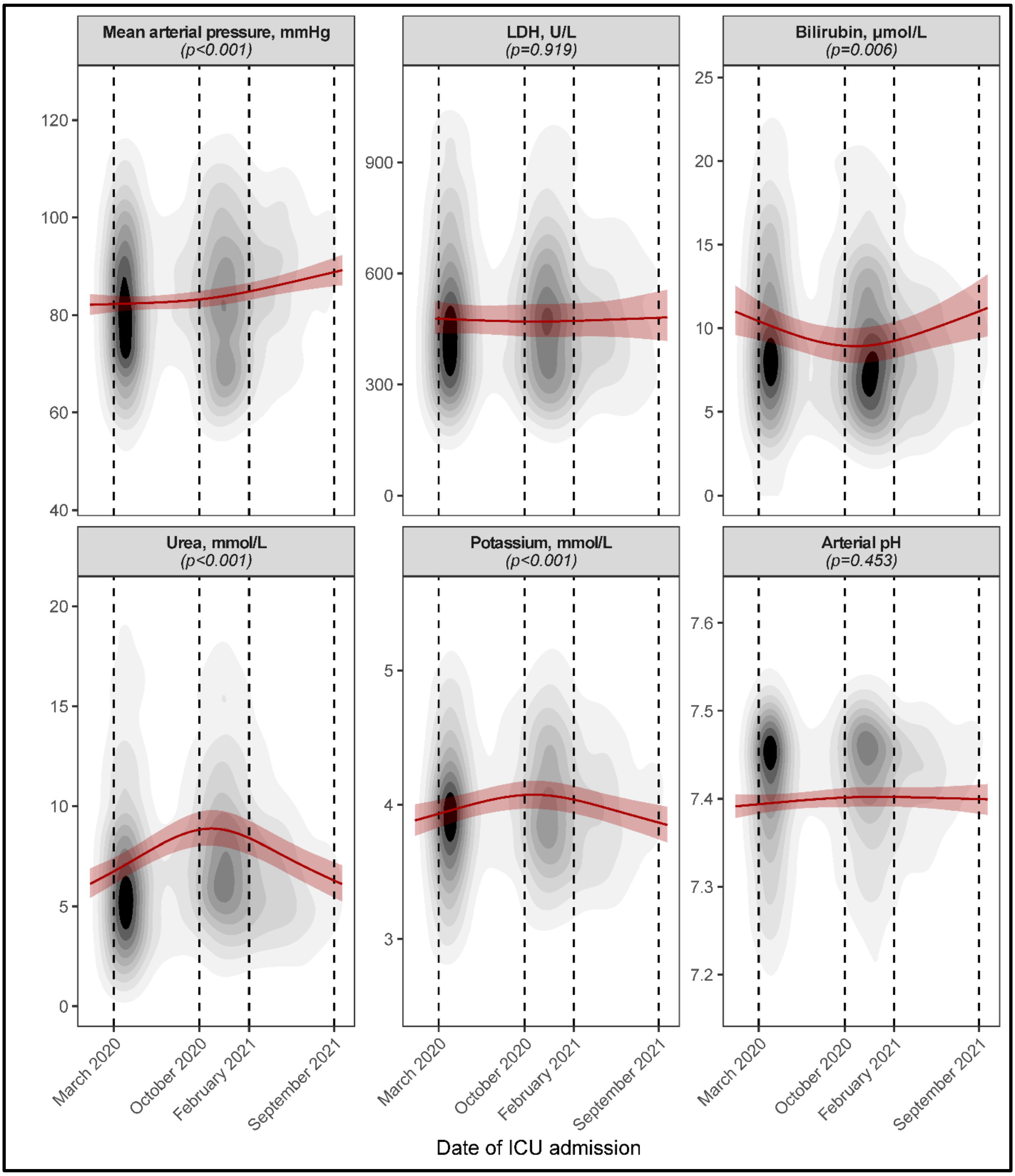

Mean effects over the time course of the pandemic, calculated by means of generalized mixed effect models, are depicted by a red continuous line. 95% confidence intervals of the effect are depicted as shaded red area. The given p-values originate from an analysis of deviance. Continuous variables are represented by topographic density plots, in which the intensity of the grayscale coloring indicates the highest concentration of values. Categorical variables are represented by violin plots, in which the segmental width of the plot correlates with the concentration of values.

e-Figure 8: Dynamics of the evolution of vital and laboratory parameters during the first five days of intensive care unit stay *(extended 1)*

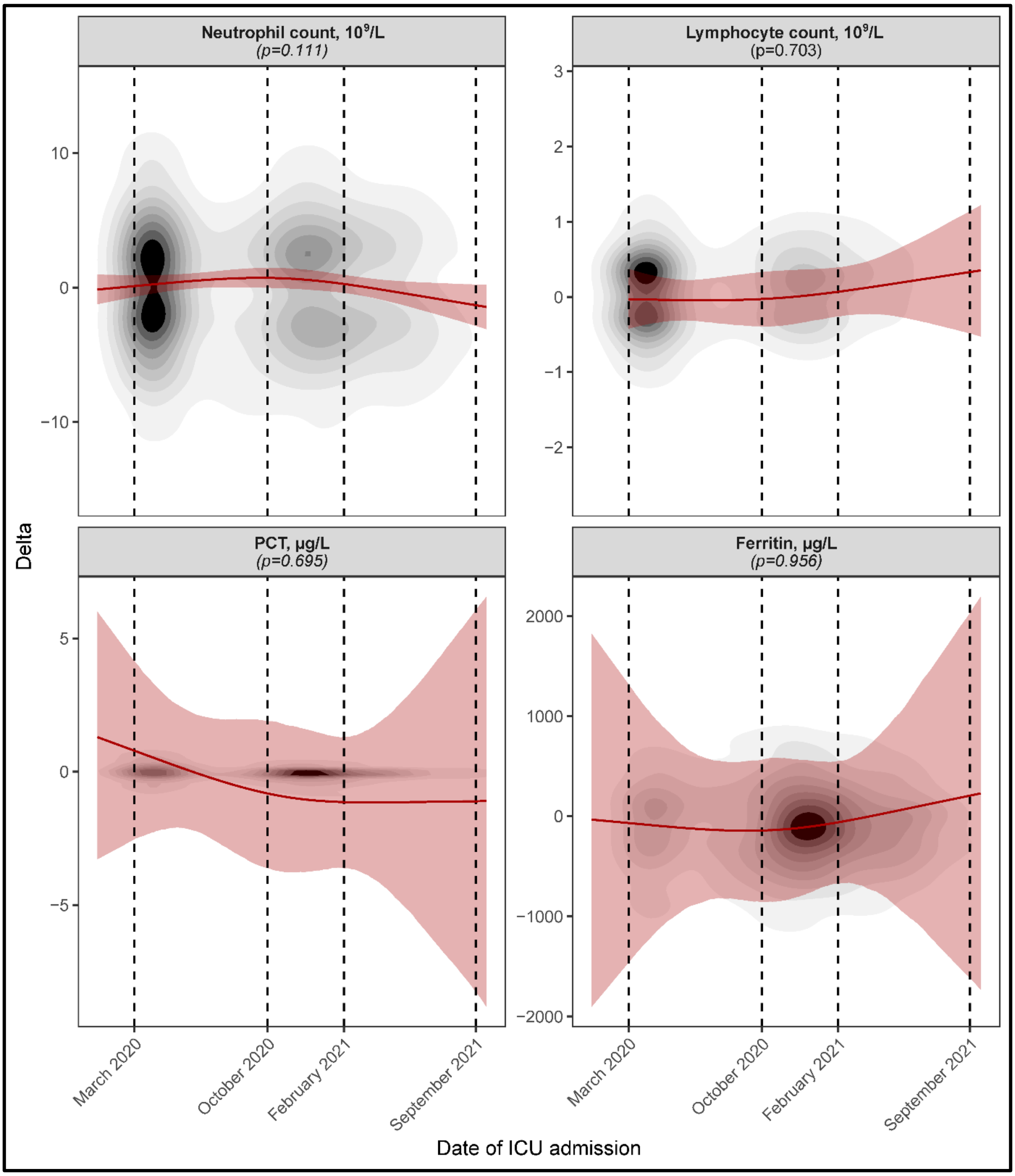

To capture the changes in the dynamics of disease over the first days of ICU stay, the difference of a variable between day 5 and day 1 is summarized as parameter (Delta) over time. Mean effects over the time course of the pandemic, calculated by means of generalized mixed effect models, are depicted by a red continuous line. 95% confidence intervals of the effect are depicted as shaded red area. The given p-values originate from an analysis of deviance. Variables are represented by topographic density plots, in which the intensity of the grayscale coloring indicates the highest concentration of values.

e-Figure 9: Dynamics of the evolution of vital and laboratory parameters during the first five days of intensive care unit stay *(extended 2)*

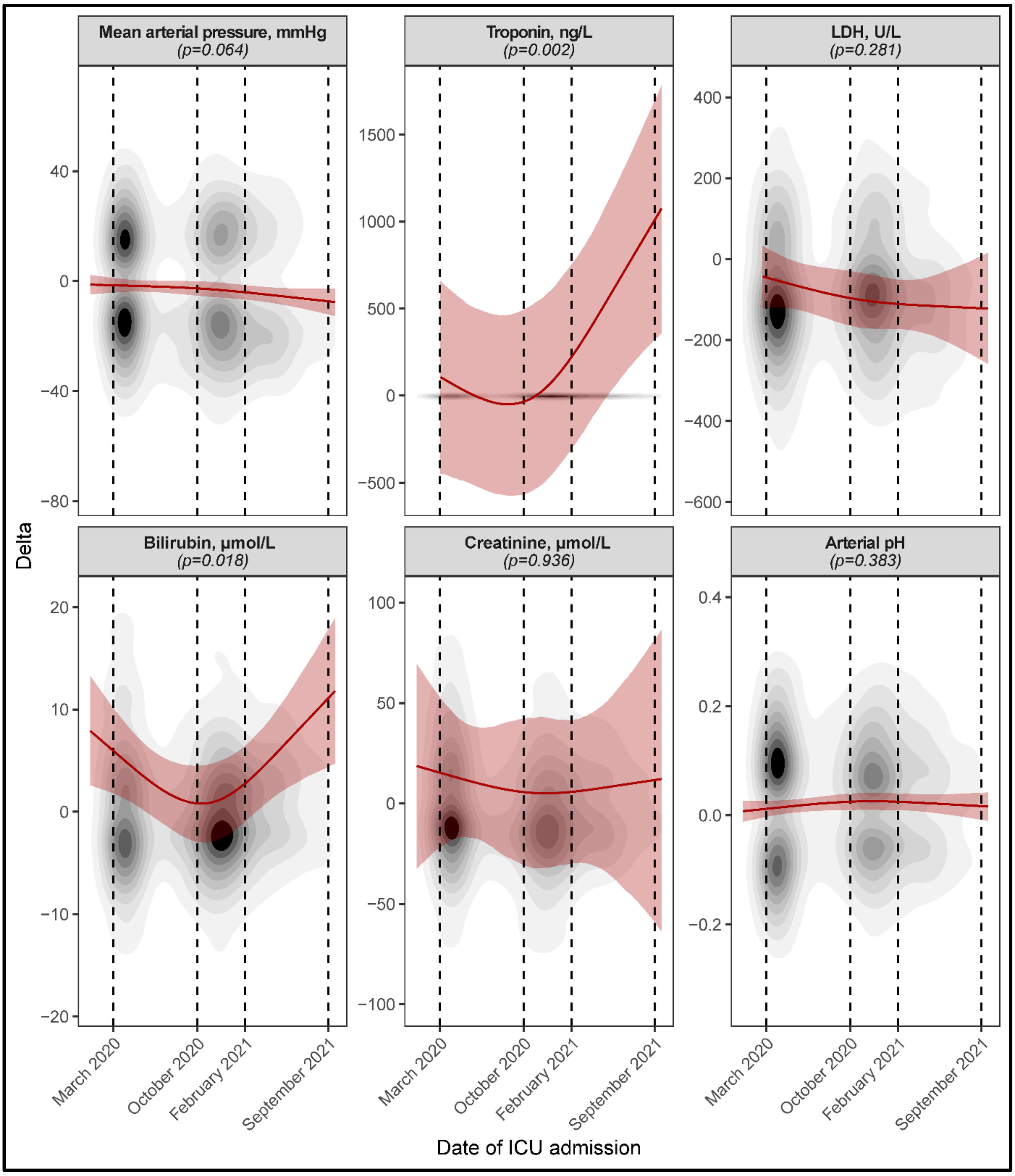

To capture the changes in the dynamics of disease over the first days of ICU stay, the difference of a variable between day 5 and day 1 is summarized as parameter (Delta) over time. Mean effects over the time course of the pandemic, calculated by means of generalized mixed effect models, are depicted by a red continuous line. 95% confidence intervals of the effect are depicted as shaded red area. The given p-values originate from an analysis of deviance. Variables are represented by topographic density plots, in which the intensity of the grayscale coloring indicates the highest concentration of values.

<u>e-Figure 10:</u> Evolution of the proportion of vaccinated patients admitted to the intensive care unit

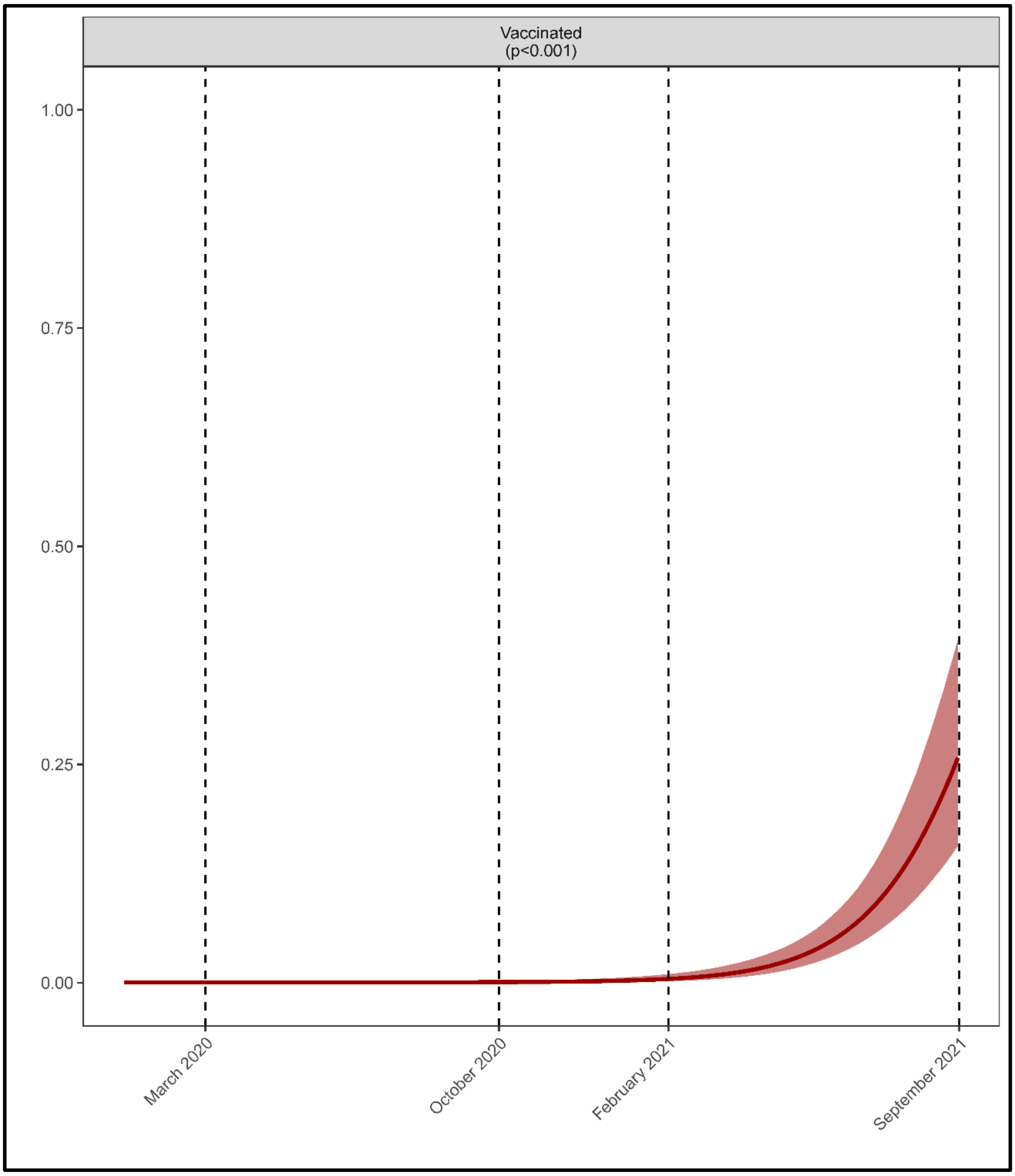

Mean effects over the time course of the pandemic, calculated by means of generalized mixed effect models, are depicted by a red continuous line. 95% confidence intervals of the effect are depicted as shaded red area. The given p-values originate from an analysis of deviance.

e-Table 2: **Dynamics of $\Delta_{late-early}$, representing the difference between day 5 and intensive care unit admission, throughout the pandemic**

	Total Population n = 4041	Mar 2020 – Sep 2020 n = 1700	Oct 2020 – Jan 2021 n = 1543	Feb 2021 – Sep 2021 n = 798
Mean arterial pressure, mmHg	-3.69 ± 27.4	-2.16 ± 24.4	-3.49 ± 27.2	-8.25 ± 33.4
Norepinephrine, µg/kg/min	-2.02 ± 18.6	-1.82 ± 23.7	-2.96 ± 11.9	-0.817 ± 11.8
Troponin, ng/L	14.3 ± 1770	46.8 ± 785	-150 ± 1610	193 ± 2660
P/F ratio, mmHg	47.0 ± 218	32.4 ± 187	27.6 ± 144	49.6 ± 173
Ventilatory ratio	-0.115 ± 1.20	0.121 ± 1.16	-0.0899 ± 1.14	-0.253 ± 1.24
White blood cell count, 10^9/L	0.439 ± 9.59	0.258 ± 9.70	1.12 ± 10.2	0.854 ± 9.02
Neutrophil count, 10^9/L	-0.102 ± 7.52	0.231 ± 7.98	0.540 ± 7.93	-0.618 ± 6.26
Lymphocyte count, 10^9/L	-0.142 ± 4.28	-0.125 ± 3.25	0.0904 ± 5.00	0.172 ± 4.36
CRP, mg/L	-78.4 ± 582	-28.0 ± 166	-74.9 ± 179	-155 ± 1330
PCT, µg/L	0.125 ± 33.9	0.257 ± 13.8	-1.54 ± 38.1	0.0873 ± 55.3
Ferritin, µg/L	-137 ± 7380	-245 ± 13300	53.5 ± 4290	-51.4 ± 1200
D-Dimers, µg/L	-163 ± 23700	908 ± 14500	-978 ± 17800	-3450 ± 45200
LDH, U/L	-85.1 ± 542	-63.7 ± 466	-96.3 ± 712	-107 ± 311
Bilirubin, µmol/L	4.07 ± 36.0	4.06 ± 23.4	1.03 ± 21.8	7.33 ± 53.7
Creatinine, µmol/L	8.11 ± 376	10.3 ± 481	5.10 ± 371	5.72 ± 108
Arterial pH	0.0321 ± 0.138	0.0281 ± 0.140	0.0311 ± 0.132	0.0281 ± 0.131

Data are presented as mean ± standard deviation. These are aggregated data and are not the result of hierarchical, generalized linear mixed-effect modelling.

<u>e-Table 3:</u> Respiratory and organ support during intensive care unit stay and outcomes *(whole population)*

	Total Population<br>n = 4041	Mar 2020 – Sep 2020<br>n = 1700	Oct 2020 – Jan 2021<br>n = 1543	Feb 2021 – Sep 2021<br>n = 798
**Respiratory support during intensive care unit stay**				
High Flow Oxygen Therapy	802 (20)	211 (12)	381 (25)	210 (26)
Non-Invasive Mechanical Ventilation	1327 (33)	405 (24)	619 (40)	303 (38)
Invasive Mechanical Ventilation	2669 (66)	1158 (72)	986 (64)	525 (66)
**Organ support during intensive care unit stay**				
Vasopressors	2350 (58)	1004 (59)	899 (58)	447 (56)
Renal Replacement Therapy	391 (10)	185 (11)	155 (10)	51 (6)
Extra-Corporeal Membrane Oxygenation	237 (6)	56 (3)	116 (8)	65 (8)
**Outcomes**				
Length of intensive care unit stay, days	14 ± 19	17 ± 21	13 ± 18	13 ± 17
Intensive care unit mortality	1063 (26)	447 (26)	452 (29)	164 (20)

Data are presented as mean ± standard deviation or counts (percentages). These are aggregated data and are not the result of hierarchical, generalized linear mixed-effect modelling.

e-Table 4: Respiratory and organ support during intensive care unit stay and outcomes *(invasive mechanically ventilated)*

	Total Population<br>n = 2669	Mar 2020 – Sep 2020<br>n = 1158	Oct 2020 – Jan 2021<br>n = 986	Feb 2021 – Sep 2021<br>n = 525
**Respiratory support during intensive care unit stay**				
High Flow Oxygen Therapy	387 (15)	101 (9)	174 (18)	112 (21)
Non-Invasive Mechanical Ventilation	882 (33)	260 (23)	409 (42)	213 (41)
Prone Position	1983 (74)	866 (75)	696 (71)	421 (80)
**Organ support during intensive care unit stay**				
Vasopressors	2207 (83)	976 (84)	814 (83)	417 (79)
Renal Replacement Therapy	386 (15)	184 (16)	152 (15)	50 (10)
Extra-Corporeal Membrane Oxygenation	234 (9)	56 (5)	114 (12)	64 (12)
**Outcomes**				
Length of intensive care unit stay, days	18 ± 21	20 ± 21	17 ± 21	16 ± 18.2
Intensive care unit mortality	905 (34)	384 (33)	387 (39)	134 (26)

Data are presented as mean ± standard deviation or counts (percentages). These are aggregated data and are not the result of hierarchical, generalized linear mixed-effect modelling.

e-Table 5: Respiratory and organ support during intensive care unit stay and outcomes *(non-invasive mechanically ventilated)*

	Total Population<br>n = 1372	Mar 2020 – Sep 2020<br>n = 542	Oct 2020 – Jan 2021<br>n = 557	Feb 2021 – Sep 2021<br>n = 273
**Respiratory support during intensive care unit stay**				
High Flow Oxygen Therapy	415 (30)	110 (20)	207 (37)	98 (36)
Non-Invasive Mechanical Ventilation	445 (32)	145 (27)	210 (38)	90 (33)
Awake Prone Position	246 (18)	64 (12)	103 (19)	79 (29)
**Organ support during intensive care unit stay**				
Vasopressors	143 (10)	28 (5)	85 (15)	30 (11)
Renal Replacement Therapy	5 (0.4)	1 (0.2)	3 (0.5)	1 (0.4)
Extra-Corporeal Membrane Oxygenation	3 (0.2)	0 (0.0)	2 (0.4)	1 (0.4)
**Outcomes**				
Length of intensive care unit stay, days	7 ± 12	8 ± 17	6 ± 9	6 ± 8
Intensive care unit mortality	139 (10)	43 (8)	66 (12)	30 (11)

Data are presented as mean ± standard deviation or counts (percentages). These are aggregated data and are not the result of hierarchical, generalized linear mixed-effect modelling.

<u>e-Figure 11:</u> Dynamics of baseline characteristics over the pandemic ***(stratified by intensive care unit survival)***

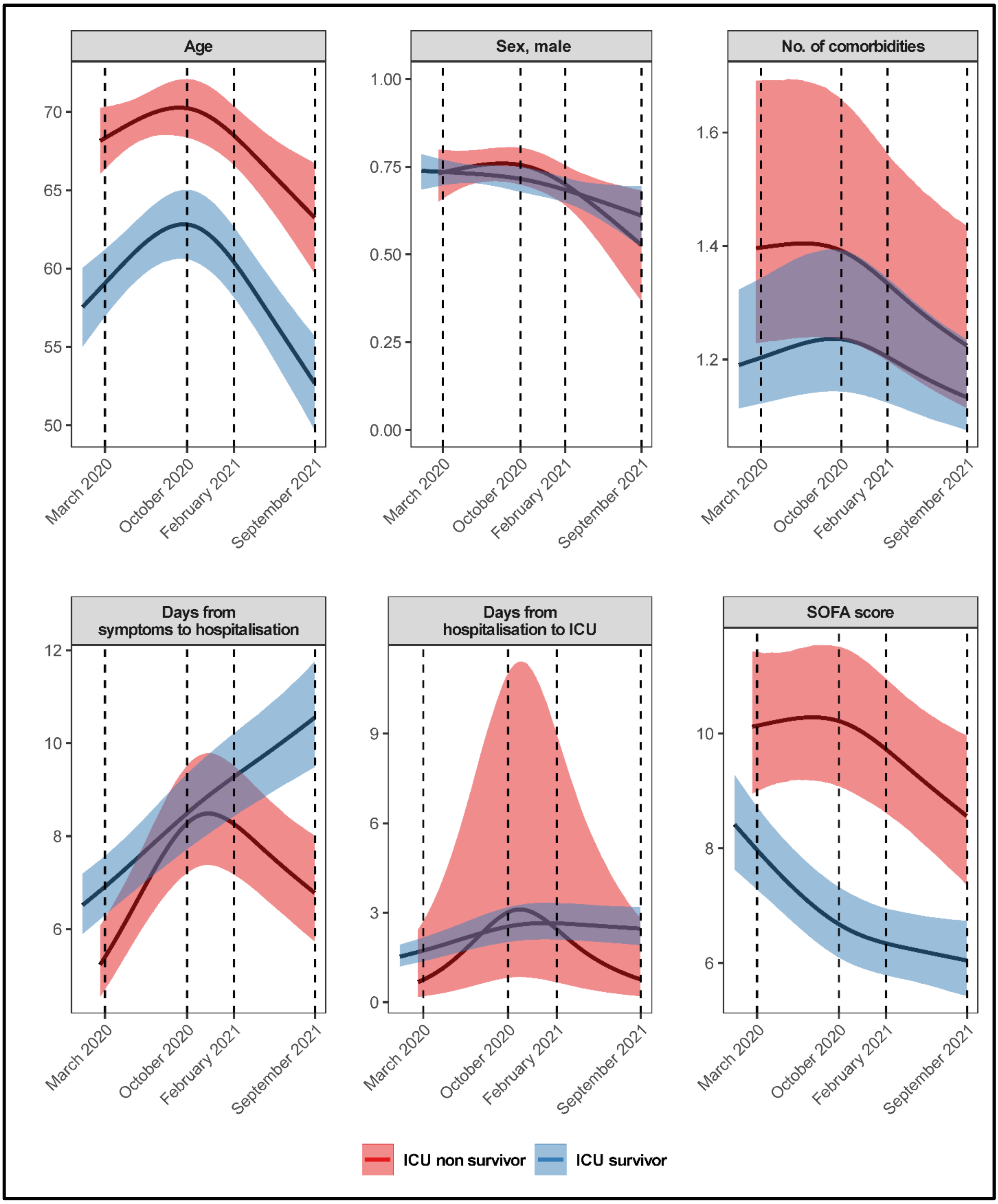

Mean effects over the time course of the pandemic, calculated by means of generalized mixed effect models, are depicted by as continuous lines. 95% confidence intervals of the effect are depicted as shaded areas. Intensive care unit survivors are colored in blue and non-survivors in red.

<u>e-Figure 12:</u> Dynamics of vitals and laboratory parameters at intensive care unit admission *(stratified by intensive care unit survival)*

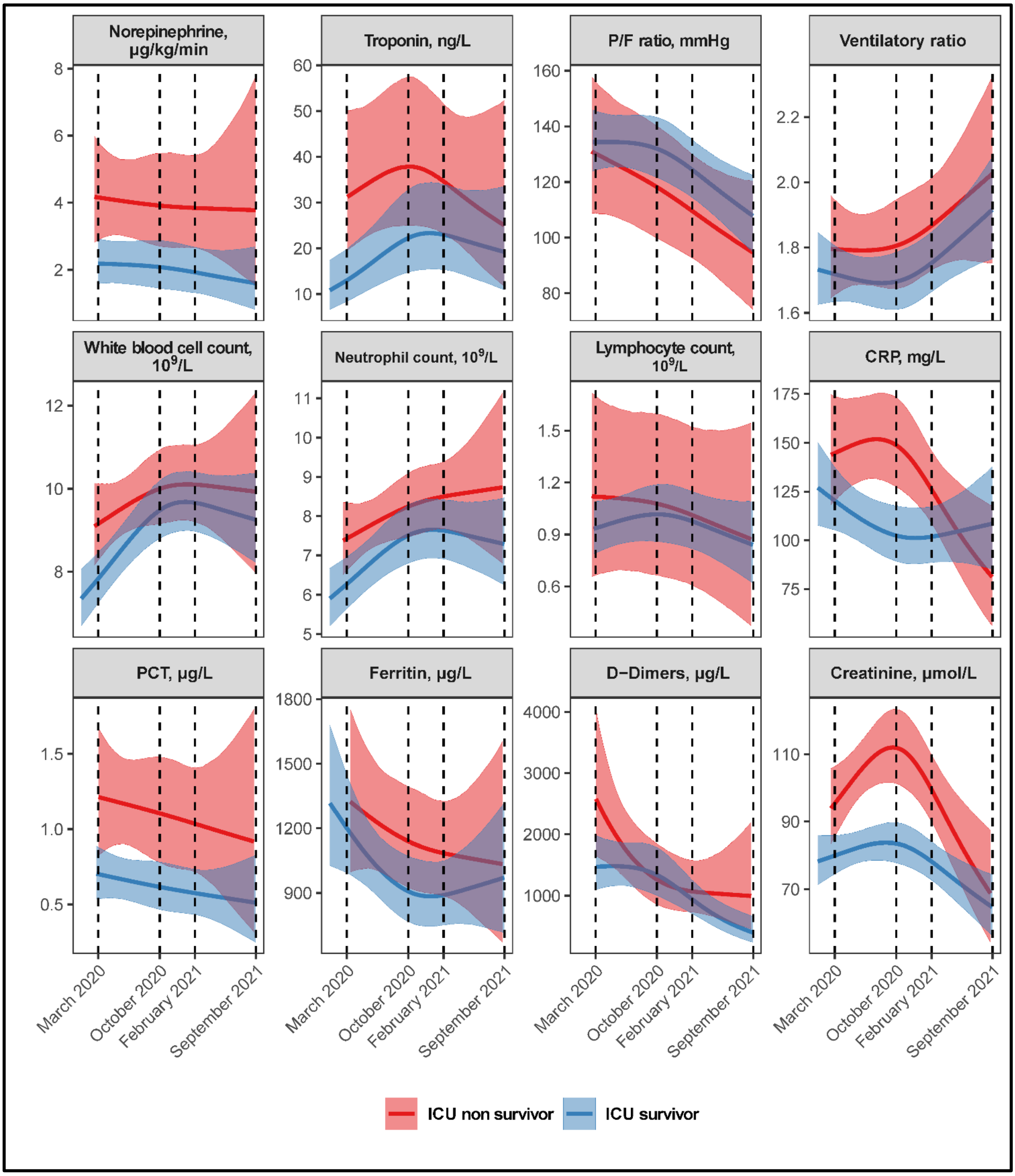

Mean effects over the time course of the pandemic, calculated by means of generalized mixed effect models, are depicted by as continuous lines. 95% confidence intervals of the effect are depicted as shaded areas. Intensive care unit survivors are colored in blue and non-survivors in red.

<u>e-Figure 13:</u> Dynamics of the evolution of vital and laboratory parameters during the first five days of intensive care unit stay *(stratified by intensive care unit survival)*

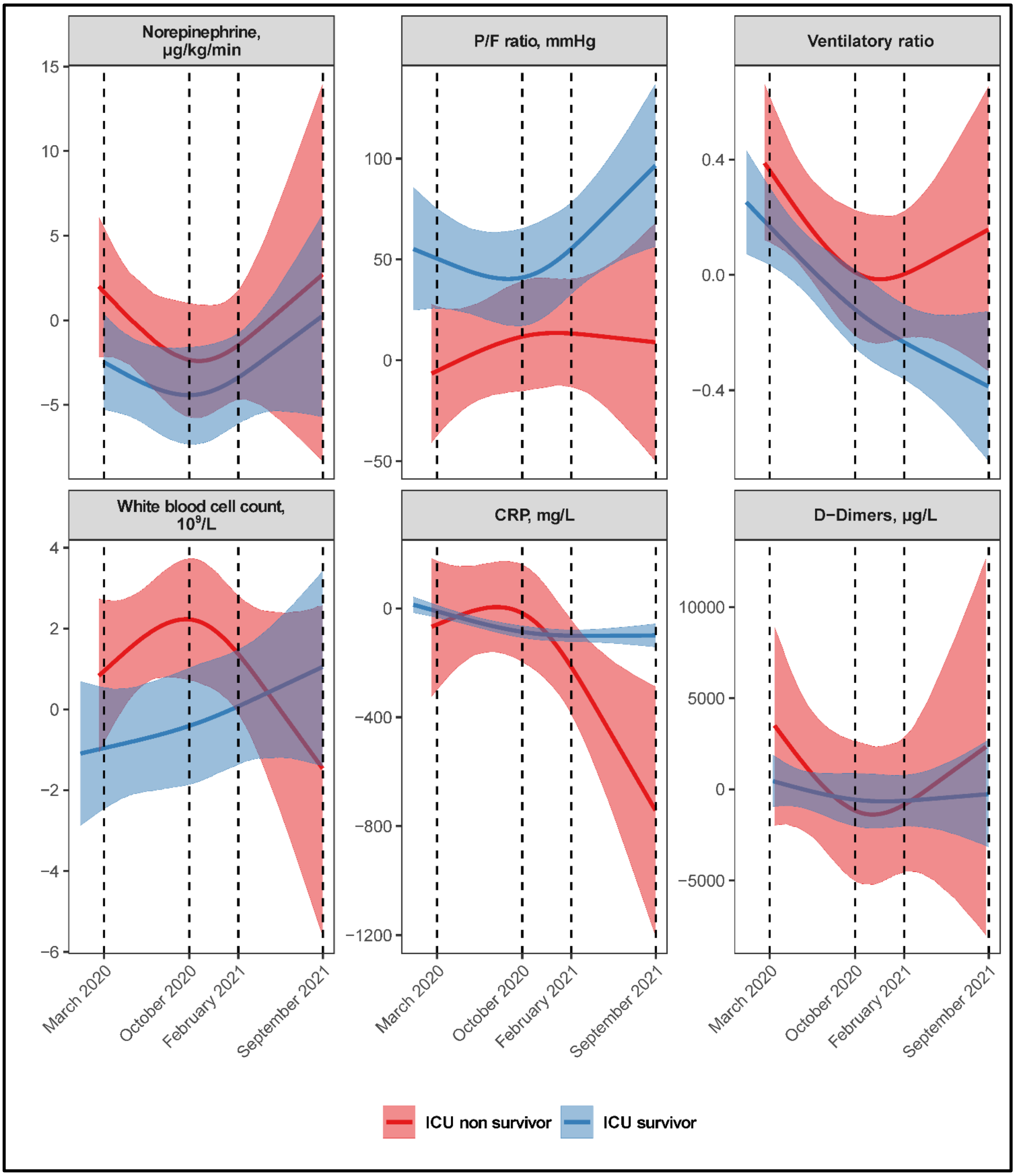

To capture the changes in the dynamics of disease over the first days of ICU stay, the difference of a variable between day 5 and day 1 is summarized as parameter (Delta) over time. Mean effects over the time course of the pandemic, calculated by means of generalized mixed effect models, are depicted by a continuous line. 95% confidence intervals of the effect are depicted as shaded area. Intensive care unit survivors are colored in blue and non-survivors in red.

e-Figure 14: **Dynamics of outcomes and organ support strategies** ***(stratified by intensive care unit survival)***

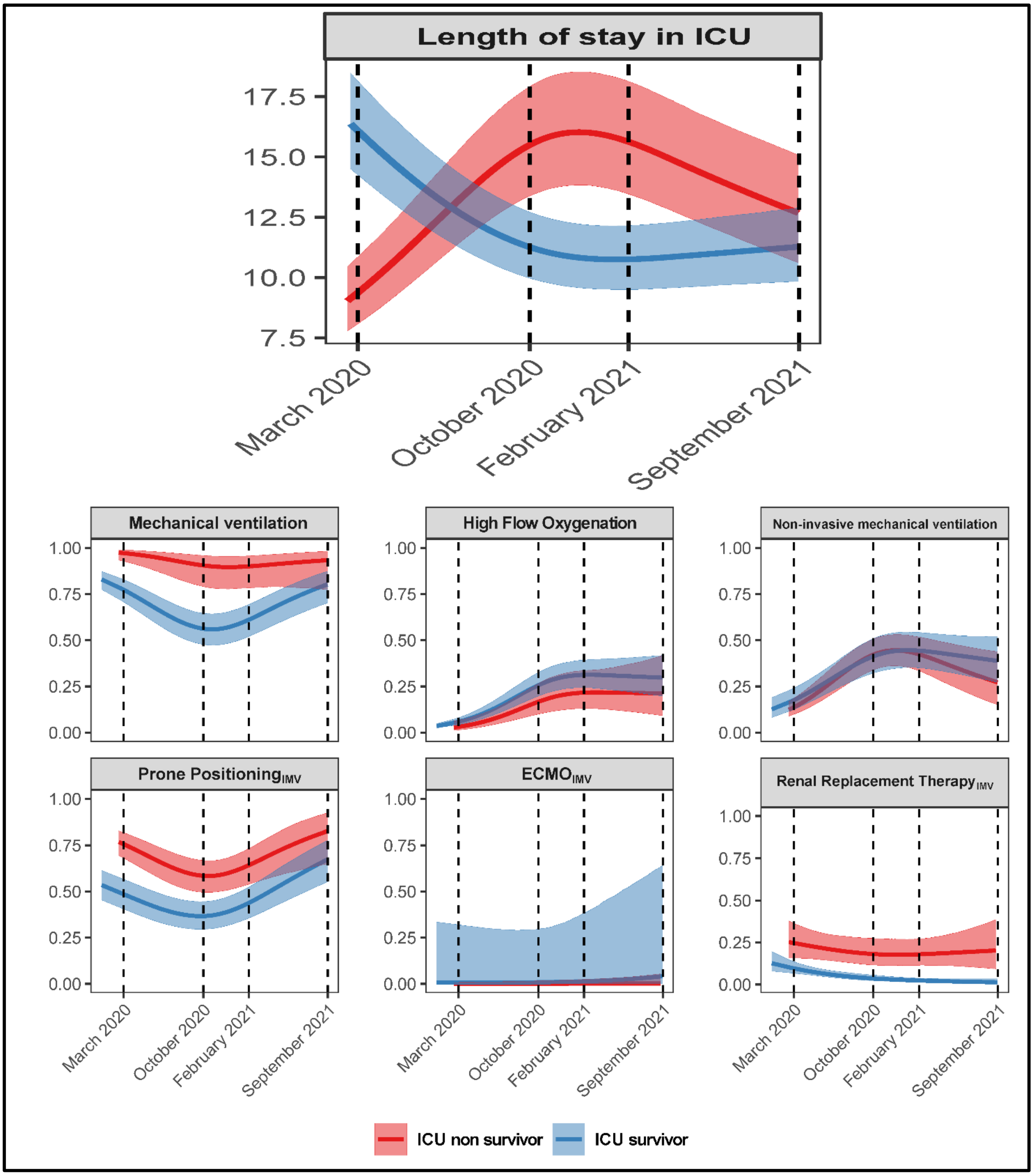

Mean effects over the time course of the pandemic, calculated by means of generalized mixed effect models, are depicted by as continuous lines. 95% confidence intervals of the effect are depicted as shaded areas. Intensive care unit survivors are colored in blue and non-survivors in red. *IMV, subgroup of patients treated with invasive mechanical ventilation.*

<u>e-Figure 15:</u> Gross domestic product per capita for selected countries participating in the registry 2020

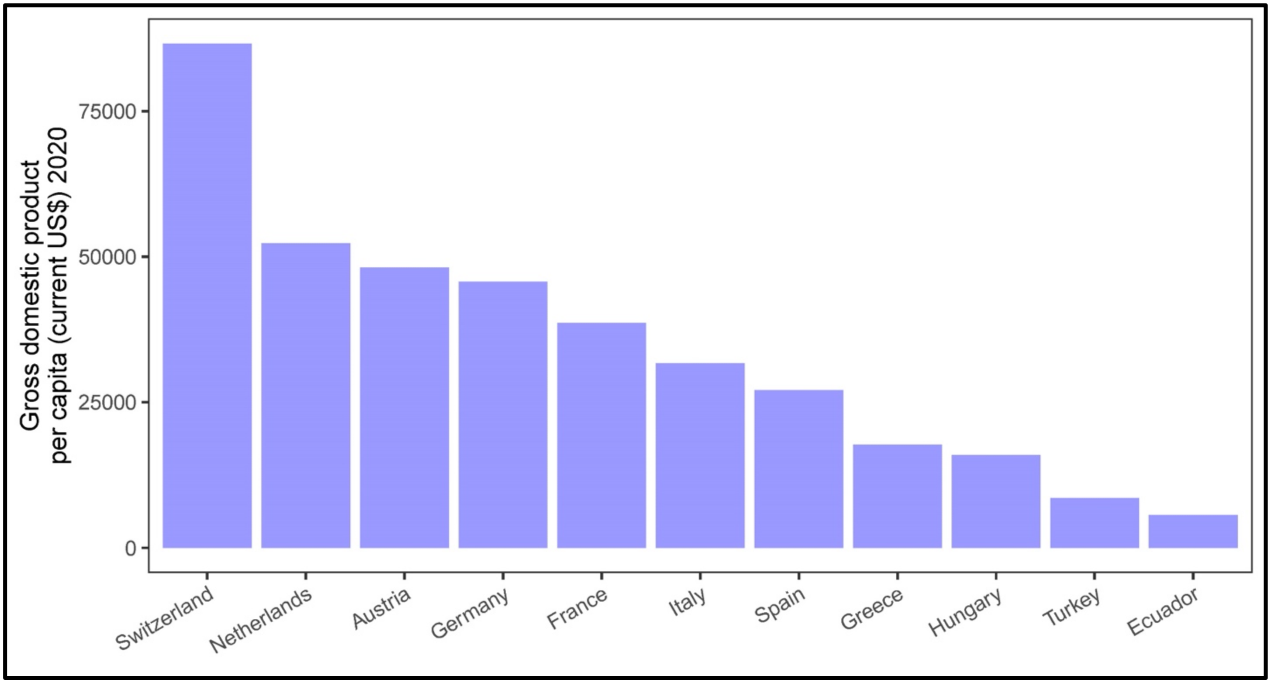

Supplement: Supplementary file 1 — Additional file 1. Online Supplementary Material. [file 13054_2022_4065_MOESM1_ESM.pdf]
